# Otterly delicious: Spatiotemporal variation in the diet of a recovering population of Eurasian otters (*Lutra lutra*) revealed through DNA metabarcoding and morphological analysis of prey remains

**DOI:** 10.1002/ece3.10038

**Published:** 2023-05-10

**Authors:** Lorna E. Drake, Jordan P. Cuff, Sergio Bedmar, Robbie McDonald, William O. C. Symondson, Elizabeth A. Chadwick

**Affiliations:** ^1^ School of Biosciences Cardiff University Cardiff UK; ^2^ School of Natural and Environmental Sciences Newcastle University Newcastle UK; ^3^ Rothamsted Insect Survey, Rothamsted Research Harpenden UK; ^4^ Department of Conservation Biology Doñana Biological Station (EBD‐CSIC) Sevilla Spain; ^5^ Environment and Sustainability Institute University of Exeter Penryn UK

**Keywords:** dietary variation, Eurasian otter, fecal analysis, food webs, hard parts analysis, trophic interactions

## Abstract

Eurasian otters are apex predators of freshwater ecosystems and a recovering species across much of their European range; investigating the dietary variation of this predator over time and space, therefore, provides opportunities to identify changes in freshwater trophic interactions and factors influencing the conservation of otter populations. Here we sampled feces from 300 dead otters across England and Wales between 2007 and 2016, conducting both morphological analyses of prey remains and dietary DNA metabarcoding. Comparison of these methods showed that greater taxonomic resolution and breadth could be achieved using DNA metabarcoding but combining data from both methodologies gave the most comprehensive dietary description. All otter demographics exploited a broad range of taxa and variation likely reflected changes in prey distributions and availability across the landscape. This study provides novel insights into the trophic generalism and adaptability of otters across Britain, which is likely to have aided their recent population recovery, and may increase their resilience to future environmental changes.

## Graphical abstract

Our national scale investigation of the trophic ecology of a recovering apex predator over a decade using molecular tools highlights that prey distributions are likely to drive such species recoveries. We show that the trophic interactions of otters vary over spatial, temporal, and biological gradients and that their trophic generalism has likely been a significant factor in their proliferation and future resilience to change. Our data highlight the strength of applying molecular and morphological approaches to dietary analysis in tandem for increased trophic network completeness.

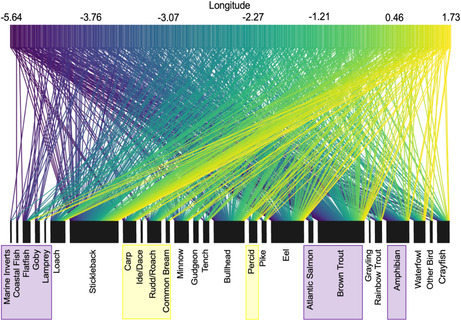

## 1 INTRODUCTION

The foraging behavior of apex predators has the potential to shape communities by directly influencing prey populations and indirectly impacting species at other trophic levels (Knight et al., 2005; Shurin et al., 2002; Wallach et al., 2015). Generalist apex predators have broad diets that span a variety of habitats and trophic levels (e.g., Berry et al., 2017; Rosenblatt et al., 2015; Vejřík et al., 2017) and tend to exhibit high levels of dietary plasticity, switching to alternative prey when their preferred prey become less available (Almeida et al., 2012; Erlinge, 1983; Murdoch, 1969; Reif et al., 2001). This plasticity makes generalist apex predators more resilient to disturbance (Peers et al., 2014; Van Baalen et al., 2001), although switching sometimes incurs fitness costs if alternative prey are nutritionally suboptimal (Cohen et al., 2014; Moorhouse‐Gann et al., 2020; Ruiz‐Olmo & Jiménez, 2009). Such dietary shifts alter the rates at which different prey species are consumed, therefore, modifying predation pressure on alternative prey species (Latham et al., 2013), which may particularly impact threatened species.

Apex predators are characteristically broadly distributed with large home ranges (Stier et al., 2016), resulting in dietary heterogeneity across broad spatiotemporal scales (Almeida et al., 2012; Lukasik & Alexander, 2011; Rosenblatt et al., 2015). Prey species differ in abundance and ease of capture between habitats and times of the year (Čech et al., 2008; Rosenblatt et al., 2015), and variation in predator diet typically reflects this (Boyd & Murray, 2001). Differences in foraging behavior between individuals can lead to differential exposure to threats, such as toxicological risk (e.g., consumption of prey species with high contaminant load) or direct mortality (e.g., due to conflict with humans associated with predation of farmed species; Stier et al., 2016). Dietary shifts can impact short‐term individual fitness (Lourenço et al., 2011; Ruiz‐Olmo & Jiménez, 2009) and the persistence of the species in the long term (Roos et al., 2001; Torres & Fonseca, 2016), consequently affecting food web dynamics and ecosystem functioning (Hollings et al., 2016; Wallach et al., 2015). This renders the assessment of apex predator trophic dynamics critical for building evidence pertinent to the conservation of both predators and prey (Gosselin et al., 2017; Pompanon et al., 2012). Dietary analysis of apex predators facilitates top‐down characterization of food webs over space and time (Bessey et al., 2019; Boyer et al., 2015). Obtaining taxonomically high‐resolution dietary data, alongside spatiotemporal and biotic data, can also elucidate pressures faced by generalist apex predators (Thomas et al., 2017) and their resilience to such pressures, allowing both individual‐ and population‐level inferences to be made (Aizpurua et al., 2018; Jeanniard‐Du‐Dot et al., 2017).

Traditionally, dietary analysis of predators has relied on the morphological identification of undigested remains in feces and stomach contents (e.g., Martins et al., 2011; McCully Phillips et al., 2019), but this is affected by several key biases. Differences in digestion rates can over‐ or underrepresent some prey, as remains that are resistant to digestion are more likely to be successfully identified (Boyer et al., 2015; Pompanon et al., 2012). Soft‐bodied prey (Arai et al., 2003), or prey that are only partially consumed (Granquist et al., 2018), are also likely to go undetected due to the lack of hard remains that can survive digestion. Where prey are morphologically similar to one another, identification can be difficult, potentially resulting in misidentified remains or poor taxonomic resolution (i.e., identified to a coarser taxonomic group; Spaulding et al., 2000). Instead, by identifying consumed prey through DNA in predator feces and stomach contents, identifications can be made to a finer taxonomic resolution even where no visual traces are present (Bowser et al., 2013; Elbrecht et al., 2017; Roslin & Majaneva, 2016; Symondson, 2002). DNA metabarcoding achieves this by combining high‐throughput sequencing (HTS) with DNA barcoding (i.e., identification of taxa by short, variable gene regions) to simultaneously identify multiple taxa within many samples (Taberlet et al., 2018). Samples can also be analyzed using multiple DNA barcoding regions targeting complementary taxa, therefore increasing the taxonomic coverage of detections (Batuecas et al., 2022; Cuff, Windsor, et al., 2022; da Silva et al., 2019; Tercel et al., 2021). Decreasing sequencing costs and the development of extensive reference databases have allowed DNA metabarcoding to be exploited by a greater range of studies (Hawlitschek et al., 2018), and it has become one of the primary methods for describing the diet of predators (e.g., Galan et al., 2018; Hardy et al., 2017; McInnes et al., 2017; Shi et al., 2018; Toju & Baba, 2018). This renders it a powerful tool for the assessment of species interactions to guide conservation management plans even over large spatiotemporal ranges.

The Eurasian otter (*Lutra lutra*, hereafter referred to as “otter”) is a generalist apex predator of European freshwater habitats, with a broad diet primarily consisting of fish (Almeida et al., 2012; Britton et al., 2006; Krawczyk et al., 2016; Kruuk, 1995). Otter population declines across much of their European range in the 1950s are generally attributed to habitat modification and acquisition of contaminants such as organochlorine pesticides and polychlorinated biphenyls (PCBs; Clavero et al., 2010; Roos et al., 2001; Strachan & Jefferies, 1996). In recent decades though, populations in Great Britain have increased and expanded their distribution, allowing otters to recolonize habitats from which they were once extirpated (Conroy & Chanin, 2002; Roos et al., 2001; Sainsbury et al., 2019). While otters have begun to return to habitats from which they have been absent in recent decades, it is likely that changes in the landscape and other factors have led to altered prey availability (Burns et al., 2016), freshwater contaminant loads (Harrad et al., 1994), and human disturbance, thereby potentially altering otter diet and foraging behavior. Corresponding changes in the health and behavior of individuals (Ruiz‐Olmo & Jiménez, 2009) are likely to alter selection pressures (Clavero et al., 2010) and thus impact the recent and continuing recovery and distribution of these populations (Stier et al., 2016). To adequately assess spatiotemporal variation in the diet of a recovering otter population, and the implications this may have for population health, accurate, and high‐throughput dietary analyses with highly resolved dietary composition data are required.

Studies of the diet of otters have primarily focused on morphological analysis of prey remains in feces or stomach contents (e.g., Almeida et al., 2012; Jędrzejewska et al., 2001; Ruiz‐Olmo & Jiménez, 2009), potentially lacking information on a range of prey species. Few studies have utilized molecular methods to analyze otter diet, with one employing DNA barcoding of prey remains (Hong et al., 2019), and six employing DNA metabarcoding (Buglione et al., 2020; Harper et al., 2020; Kumari et al., 2019; Marcolin et al., 2020; Martínez‐Abraín et al., 2020; Pertoldi et al., 2021). DNA studies into otter diet have, however, been limited either by small sample size (Hong et al., 2019, *n* = 24; Kumari et al., 2019, *n* = 7; Buglione et al., 2020, *n* = 51; Marcolin et al., 2020, *n* = 50; Martínez‐Abraín et al., 2020 *n* = 50; Pertoldi et al., 2021, *n* = 54) or use of only one barcoding region (Hong et al., 2019, 12S vertebrate‐specific; Buglione et al., 2020, 16S vertebrate‐specific; Harper et al., 2020, 12S vertebrate‐specific; Marcolin et al., 2020, 18S V9 region; Martínez‐Abraín et al., 2020, 12S teleost‐specific; Pertoldi et al., 2021, 12S vertebrate‐specific), potentially missing prey items due to primer bias or poor reference databases (Harper et al., 2020; Marcolin et al., 2020; Pertoldi et al., 2021).

Here, we used multi‐marker DNA metabarcoding alongside morphological analysis of undigested remains to assess the spatiotemporal dynamics of otter trophic interactions on a national scale. We also provide a direct comparison of DNA metabarcoding and hard‐parts analysis for the dietary characterization of otters. We tested the following hypotheses: (i) DNA metabarcoding detects a greater range of prey and identifies prey to a finer taxonomic resolution than morphological analysis, (ii) the composition of otter diet varies across landscape gradients, likely reflecting changes in prey availability, (iii) the composition of otter diet varies over seasonal and annual timescales, likely reflecting temporal changes in prey availability, (iv) dietary composition varies between different demographic groups, and (v) body condition is associated with the dietary variation, with individuals with better body condition consuming a distinct range of species, likely related to nutritional benefits.

## 2 METHODS

### 2.1 Sample and data collection

Samples and associated metadata were acquired from 300 otters collected between 2007 and 2016, obtained from the Cardiff University Otter Project, a national monitoring program for dead otters sampled from across Great Britain (https://www.cardiff.ac.uk/otter‐project). Most otters collected were killed by road traffic accidents, with a minority dying through drowning, being shot, starvation, or disease. Information on date (year and month) and location (as grid reference) of carcass collection were recorded at the site of collection. Grid references were used to plot data for spatial analysis. Detailed postmortems were performed for each carcass during which biotic data were obtained (e.g., sex and size of an individual). Fecal samples were collected from the rectum during postmortem examination, wrapped in foil, and stored at −20°C.

Following postmortems, the scaled mass index (SMI) was calculated for each individual otter using the following equation (Peig & Green, 2009, 2010):

$$\mathrm{SMI}=M_{i}{\left[L_{0}/L_{i}\;\right]}^{\text{bSMA}},$$

where *M*_*i*_ is the body mass and *L*_*i*_ is the length measurement of individual *i*, *L*_0_ is the mean length measurement for the entire study population, and bSMA is the scaling exponent. The length was measured from nose to tail‐tip to the nearest 5 mm. The mean length and the scaling exponent were both calculated from all otter data available as of January 2017 (*n* = 2477). The scaling exponent is the slope from the standard major axis regression of log‐transformed values of mass against length.

Otters were also classified into size categories based on their total length (nose to tail tip) using the “bins” function in R (OneR v2.2 package; von Jouanne‐Diedrich, 2017), which applies a clustering method using Jenks natural breaks optimization. Male and female otters were clustered separately into small (males <1046 mm, females <936 mm long), medium (males between 1046 mm and 1131 mm, females between 936 mm and 1031 mm), and large (males >1131 mm, females >1031 mm).

### 2.2 Spatial data

Spatial data describing proximity to the coast, urban land‐use, altitude, slope, and primary water habitat were collated using QGIS version 3.4.4 (QGIS Development Team, 2019). Distance from the coast was calculated as the shortest distance (km) along a river from the location at which the otter was found to the low tide point of the mouth of the river (hereafter referred to as “river distance”), using the package RivEX (Hornby, 2020), because otters tend to travel along water courses rather than across land. As most otters were found as roadkill, and not all were adjacent to rivers, each otter was first assigned to the nearest river. Locations more than 1000 m from a river were checked, and if there was more than one river along which the otter might have traveled, then river distance was calculated for all rivers, and a mean distance was used. All otters within 1000 m of the coast were given a distance of zero if they were closer to the coastline than a river.

Otter locations were mapped as points, and circular areas of 10 km diameter (hereafter referred to as “buffers”) were mapped around each. Fecal samples typically reflect diet from the preceding 24–72 h (in mammals; Casper et al., 2007; Deagle et al., 2005; Thalinger et al., 2016), during which time otters can travel up to 10 km (Chanin, 2003), it was therefore deemed appropriate to use this distance to reflect the land used by otters within the sample timeframe. Buffers were used to calculate proportions of urban land‐use (i.e., urban and suburban land‐use extracted from the 25 m resolution UK land cover map from 2007; Morton et al., 2011), mean altitude, and mean slope (extracted from European Digital Elevation Model [EU‐DEM] map; European Environment Agency, 2011). We chose to focus on urban land‐use as urbanization may affect otter diets either through changes to prey assemblages or disturbance affecting an otter's ability to forage. Longitude, altitude, and slope were highly correlated (Figure S1), therefore, longitude was used in further analyses as a representative for the three variables.

Otters in England and Wales typically feed in freshwater river systems but will opportunistically feed in lakes or at the coast if these habitats are within range (Clavero et al., 2004; Jędrzejewska et al., 2001; Parry et al., 2011). Available prey differs between lakes, coasts, and river systems as well as between different parts of the river network (e.g., tributary, main river channel). To assess whether water habitat type influenced dietary variation, we designated each otter to one of the following: transitional water (coastal and estuarine), lake, main river channel, or tributary (based on Water Framework Directive 2000/60/EC designations mapped using GIS shapefiles provided by Natural Resources Wales and Environment Agency). Otters within 2.5 km (half of a buffer's radius) from a lake or transitional water were assigned to that habitat, while those further away were assumed to be feeding primarily in the river network. The RivEX network map (Hornby, 2020) was used to map all rivers, and individuals were further categorized according to whether their assumed habitat was primarily main river or tributary. To do this, the total length of main river channels and tributaries was calculated within each 10 km buffer. The length of main channels was weighted 10 times greater to account for the greater cross‐section of a main channel compared with tributaries (Benda et al., 2004) because waterways with greater areas are assumed to support more prey (Samarasin et al., 2014). The sum of weighted main river lengths and tributary lengths was calculated, and if more than 50% of each buffer was attributed to the main river channel, the otter was assigned to the main river channel, otherwise, it was assigned to the tributary.

### 2.3 Morphological analysis

Each fecal sample was first thawed, homogenized by hand in a sterile container, and divided into subsamples; three samples weighing 200 mg each were collected for DNA analysis (one sample used for DNA extraction and the other two frozen as back‐ups), and the remaining material was used for morphological analysis. Subsamples undergoing morphological analysis were then soaked in a solution of water and commercial liquid biological detergent (water:detergent, 10:1) for 24 h. Samples were passed through sieves with a 0.5 mm mesh and washed with water to ensure only hard parts remained which were air‐dried for 24 h. A record was made of any samples that did not contain any hard parts. Recognizable remains (bones, fish scales, feathers, and fur) underwent microscopic identification using a range of keys (Coburn & Gaglione, 1992; Conroy et al., 2005; Libois & Hallet‐Libois, 1987; Miranda & Escala 2002; Prenda & Granado‐Lorencio, 1992; Prenda et al., 1997; Tercerie et al., 2019; University of Nottingham, 2020; Watt et al., 1997). Prey remains were identified to the finest possible taxonomic resolution and recorded as present within or absent from a sample.

### 2.4 DNA metabarcoding analysis

Fecal samples were processed for HTS, and subsequent bioinformatic analysis was conducted, as described in Drake et al. (2022, also described in detail in Appendix S1.1–S1.5; Figure S2). In summary, DNA was extracted from a subsample of fecal material and amplified using two metabarcoding primer pairs, designed to amplify regions of the 16S rRNA and cytochrome *c* oxidase subunit I (COI) genes, each primer having 10 base pair molecular identifier tags (MID tags) to facilitate postbioinformatic sample identification.

Two primer pairs from different gene regions were selected to overcome biases associated with each region and broaden the range of taxa amplified: the 16S barcoding region targeted for vertebrate DNA and cytochrome *c* oxidase subunit I (COI) for invertebrate DNA. For 16S, the novel primer pair FN2199 (5′‐yayaagacgagaagaccct‐3′) and R8B7 (5′‐ttatccctrgggtarcthgg‐3′; modified for this study from Deagle et al., 2009) were used, which targeted a 186–228 bp amplicon. For COI, the primer pair Mod_mCOIintF (5′‐ggwacwggwtgaacwgtwtaycc‐3′; modified for this study from Leray et al., 2013) and HCO2198 (5′‐taaacttcagggtgaccaaaaaatca‐3′; Folmer et al., 1994) were used, which targeted a 316 bp amplicon. Likelihood of amplification of target otter prey taxa was determined via in silico testing using ecoPCR (Boyer et al., 2016) and confirmed in vitro via PCR under the conditions used for the final assays (Appendix S1.2) with otters and known prey DNA. In silico and in vitro tests demonstrated that both primer pairs amplified target taxa. COI primers also amplified some vertebrate taxa despite being targeted at invertebrates but did so with reduced coverage compared with vertebrate‐targeted 16S primers.

Fecal samples were processed alongside extraction and PCR‐negative controls, repeat samples, and mock communities, which comprised standardized mixtures of DNA of marine species not previously detected in the diet of Eurasian otters, and some tag combinations were left unused to identify tag jumping. The resultant DNA libraries for each marker were sequenced on separate MiSeq V2 chips with 2 × 250 bp paired‐end reads. Bioinformatic analyses were carried out using a custom pipeline, following which sequencing data underwent filtering steps to remove any remaining artifacts or contaminants in the data (Drake et al., 2022). Filtering involved removing taxa from each sample that contributed less than 0.5% of a sample's total reads for 16S and 0.3% for COI. Reads equal to or less than the maximum read count identified in unused MID–tag combinations or negative controls per taxon were also removed. This method was conservative but was selected to remove false positives which would otherwise overrepresent some prey groups present in some samples as contaminants (Drake et al., 2022).

Reads were assigned to the finest possible taxonomic resolution and recorded as present within or absent from a sample, separately for 16S and COI. Reads assigned to nonfood items remaining in the analysis were removed, these included taxa not assigned to the animal kingdom (e.g., fungi and bacteria, which were not considered pertinent to this study), those with poor taxonomic resolution (e.g., Eutheria, which includes all extant British mammals and thus was not useful for further analyses), reads from otters themselves (e.g., those assigned to *Lutra lutra*; Cuff, Kitson, et al., 2022) and taxa with a maximum size <3 mm (e.g., diatoms, assumed to be due to secondary or accidental predation). Following the removal of nonfood items, data from the two data sets were combined to give a more complete representation of the diet of otters through the complementarity of the separate taxonomic biases of the two primer pairs. If a taxon was present in either of the metabarcoding data sets, then that taxon was recorded as present in that sample. If a prey item was detected in a sample in both metabarcoding data sets, but at different levels of taxonomic resolution, only the presence with the finer taxonomic resolution was retained.

### 2.5 Comparison of methods

The frequency of occurrence for each prey item detected across the 300 otters screened was calculated for both morphological and metabarcoding data sets, allowing the two methods to be directly compared. Presences assigned to “insect,” “beetle,” “mollusk,” and “snail” in the morphological analysis were removed before comparing data sets; many identifications from these particular taxonomic groups were identified to a finer resolution through metabarcoding but removed as secondary predation or accidental consumption (Tercel et al., 2021), therefore, these presences in the morphological analysis were also deemed likely to have occurred through secondary predation. Presences assigned to “mammal” (identified from fur) in the morphological analysis were also removed before comparing data sets due to the uncertainty of fur coming from the otter grooming itself and metabarcoding identifying the otter as the only mammal in these samples. Presence–absence matrices produced from each methodology were also combined in order to assess the overlap in data. Where both methods identified the same taxonomic group, the presence was assigned to the finest taxonomic resolution, whereas where there was ambiguity about whether the methods were detecting the same taxon or not, the presence was assigned to a coarser taxon. Combining data sets from each method revealed which data points were only detected by one method and which were detected by both (either at the same taxonomic level or at different resolutions). Binary matrices for prey detections were combined for the two data types, but each sample was represented separately for each method (i.e., not aggregated by sample).

### 2.6 Statistical analysis

The association between otter diet composition and biotic and abiotic drivers was explored using the combined data from morphological analysis and metabarcoding. Each taxon was assigned to a “prey group” based on similarities in taxonomy, morphology, and ecological niche (Table S5). A small number of prey identified to coarse taxonomic levels could not be assigned to a group and were removed from subsequent analyses (prey presences removed: “*Salmo* sp.,” *n* = 5; “Cyprinid,” *n* = 2; “Bird,” *n* = 2). A prey group was recorded as present in an individual fecal sample if any one (or more) of the taxa assigned to that group were present. If a prey group occurred in less than three samples, then the prey group was designated as rare and removed from subsequent analyses (Table S5). Dietary composition was compared against biotic and abiotic drivers via multivariate generalized linear models in R (version 3.6.0) and R Studio (version 1.2.1335) (R Core Team, 2019) using “mvabund” and visualized using “bipartitie” and “boral” packages (Appendix S2.4–S2.6).

The mvabund package allows model‐based analysis of multivariate data to test hypotheses regarding the effects of environmental variables on the composition of dietary data (Wang et al., 2012). Multivariate generalized linear models (MGLMs) are a robust method for detecting differences in communities with less abundant taxa and are less prone to misinterpretations due to mean–variance effects, compared to distance‐based methods (Warton et al., 2012). The “many.glm” function was used to create an MGLM using a binomial family and a “cloglog” link function. The global models included the following fixed variables: sex, size of otter, SMI, year, season, distance from the coast (km), primary water habitat, percentage of urban land‐use, latitude, and longitude (Table S1). Interactions between sex and size of otter, distance from the coast and sex, distance from the coast and size, primary water habitat and sex, primary water habitat and size, and between latitude and longitude were also included in the global model. Model assumptions were checked on the global model before conducting model selection via Akaike's Information Criterion (AIC) using the stepwise algorithm in the step function (Hastie & Pregibon, 1992; Venables & Ripley, 2002). The final model included the fixed variables longitude and distance from the coast. The significance of fixed variables on the overall diet and for specific prey groups was determined via likelihood ratio test using the “anova.manyglm” function with Monte Carlo resampling and corrected univariate *p* values for multiple testing.

To complement the mvabund analysis, the boral package was used to plot significant variables. The boral package conducts Bayesian ordination and regression analysis on multivariate data (Hui, 2016). Binomial models for boral analysis included the same fixed and response variables as in the final mvabund model. The number of latent variables was set as two. Model assumptions were checked, and latent variable values were extracted. Latent variables were plotted against significant fixed variables to visualize the individual samples and the indicator species that best described their position in a low‐dimension ordination plot. Bipartite network plots were also created to visualize the structure and identity of otter trophic interactions against significant fixed variables using the plotweb function in the bipartite package (Dormann et al., 2008). Data generated by both metabarcoding and hard‐parts analysis were visually compared using nonmetric multidimensional scaling via the “metaMDS” command with a Jaccard distance matrix and 999 tries in the “vegan” package (Oksanen et al., 2016). Only samples for which both metabarcoding and hard‐parts data were available were included in these plots.

## 3 RESULTS

Otters consumed a wide range of vertebrate and invertebrate taxa (66 vertebrate and 16 invertebrate taxa; Tables S2–S5; Figures S3 and S4). Vertebrate prey taxa primarily consisted of freshwater fish, but amphibians, birds (primarily waterfowl), mammals, and coastal fish were also identified. Invertebrate prey taxa primarily consisted of crayfish, with some mollusks, insects, earthworms, and marine invertebrates also identified at low frequencies. Taxonomic classifications within each prey group varied between morphological and metabarcoding analyses.

### 3.1 Morphological analysis

Of the 300 otters screened, morphological analysis recovered 279 occurrences of prey from 23 taxa in 172 otters, with a mean of 1.62 taxa per otter. Dietary data were not recovered from 128 otters due to the absence of hard parts suitable for morphological analysis, prey remains being assigned to secondary prey items or due to poor taxonomic resolution. Of the taxa detected, 22 were identified as vertebrates (11 to species level, eight to family, two to order, and one to class) and one was identified as an invertebrate (family level describing crayfish, Astacidae).

### 3.2 DNA metabarcoding analysis

Sequencing yielded 17.6 million paired‐end reads for the 16S library and 13.7 million for the COI library, which was reduced to 5 million for 16S and 1.1 million for COI following data processing (Figure S5). Of the 300 otters screened, dietary data were recovered for 241 otters using 16S, with a mean of 20,618 reads and 2.87 taxa per otter, and 149 using COI, with a mean of 7509 reads and 1.6 taxa per otter. Dietary data were not recovered in 42 otters due to poor amplification of DNA, DNA being assigned to nonfood items or due to poor taxonomic resolution. Retained reads were assigned to 54 vertebrate taxa (48 to species level, one to genus, and four to family) in the 16S data, while COI data were assigned to 21 vertebrate taxa (18 to species level, one to genus, and one to family) and 15 invertebrate taxa (14 to species level and one to genus). Combined results from metabarcoding data sets produced 799 occurrences of prey from 70 tax in 258 otters, with a mean of 3.08 taxa per otter. There were 567 occurrences and 34 taxa only detected using 16S primers, 109 occurrences, and 17 taxa only detected using COI primers, and 123 occurrences and 18 taxa detected using both primer sets.

### 3.3 Comparison of methods

**FIGURE 1:**
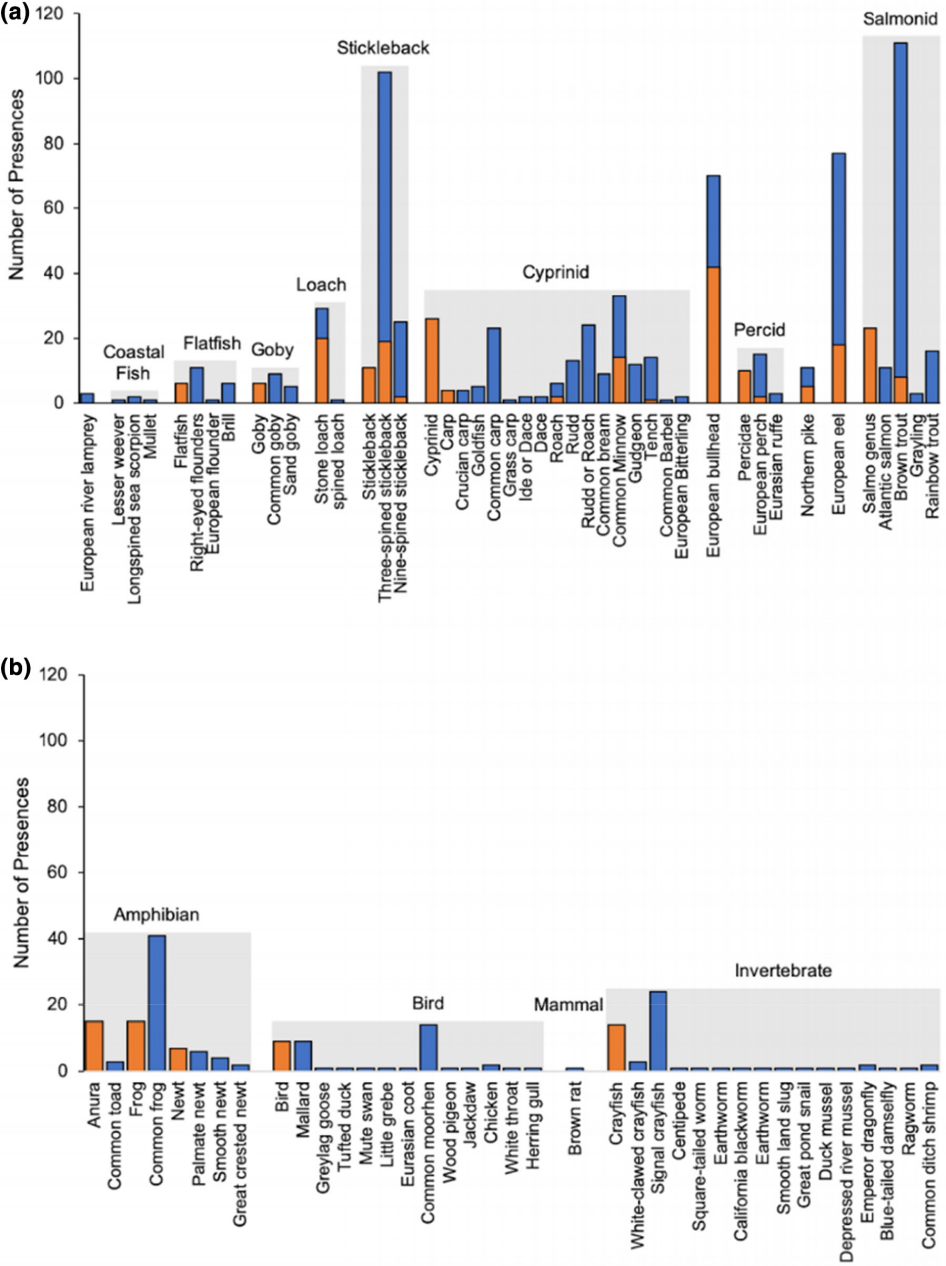
Taxon presence in the diet of Eurasian otters (*Lutra lutra*) were identified using morphological analysis of prey remains (orange) and DNA metabarcoding (blue) on fecal samples. Gray boxes show similar taxonomic groups from (a) fish and (b) other prey groups. Fecal samples were obtained from dead otters collected from across England and Wales from 2007 to 2016.

Dietary data were recovered for 268/300 otters in total; prey items were identified only by morphological analysis from 10 otters, only by metabarcoding from 96 otters, and by both methods from 162 otters. Following the removal of suspected secondary prey items, metabarcoding identified 20 taxa that were not detected using morphological analysis, 39 taxa were identified to a greater resolution by metabarcoding, and 11 taxa were identified to the same taxonomic level using both methods (Figure 1). Of the nine taxa only identified by morphological analysis, all were identified by metabarcoding at a greater taxonomic resolution (e.g., where the morphological analysis identified crayfish to genus level, metabarcoding instead identified two separate species of crayfish; Figure 1). Metabarcoding identified 528 prey item presences that were not detected using morphological analysis, 144 presences were detected at a greater resolution by metabarcoding, and 122 were identified to the same taxonomic resolution using both methods. The morphological analysis detected 45 prey item presences that were not detected by metabarcoding, but only detected one presence to a greater taxonomic resolution (one metabarcoding identification of “rudd/roach” was distinguished to “rudd” using morphological analysis). Taxa that were identified by both methods were detected at a greater frequency of occurrence using metabarcoding. The frequency of occurrence of each prey group differed with the method of dietary analysis: based on morphological analysis, bullhead was the most frequently detected taxon (14%), followed by amphibians (12%) and stickleback (11%) and based on metabarcoding, brown trout and stickleback were the most frequently detected taxa (both at 37%), followed by eel (27%) and bullhead (23%; Figure 1). The dietary compositions determined using each method were often less variable between samples than between methods (Figure S6).

### 3.4 Dietary variation

**FIGURE 2:**
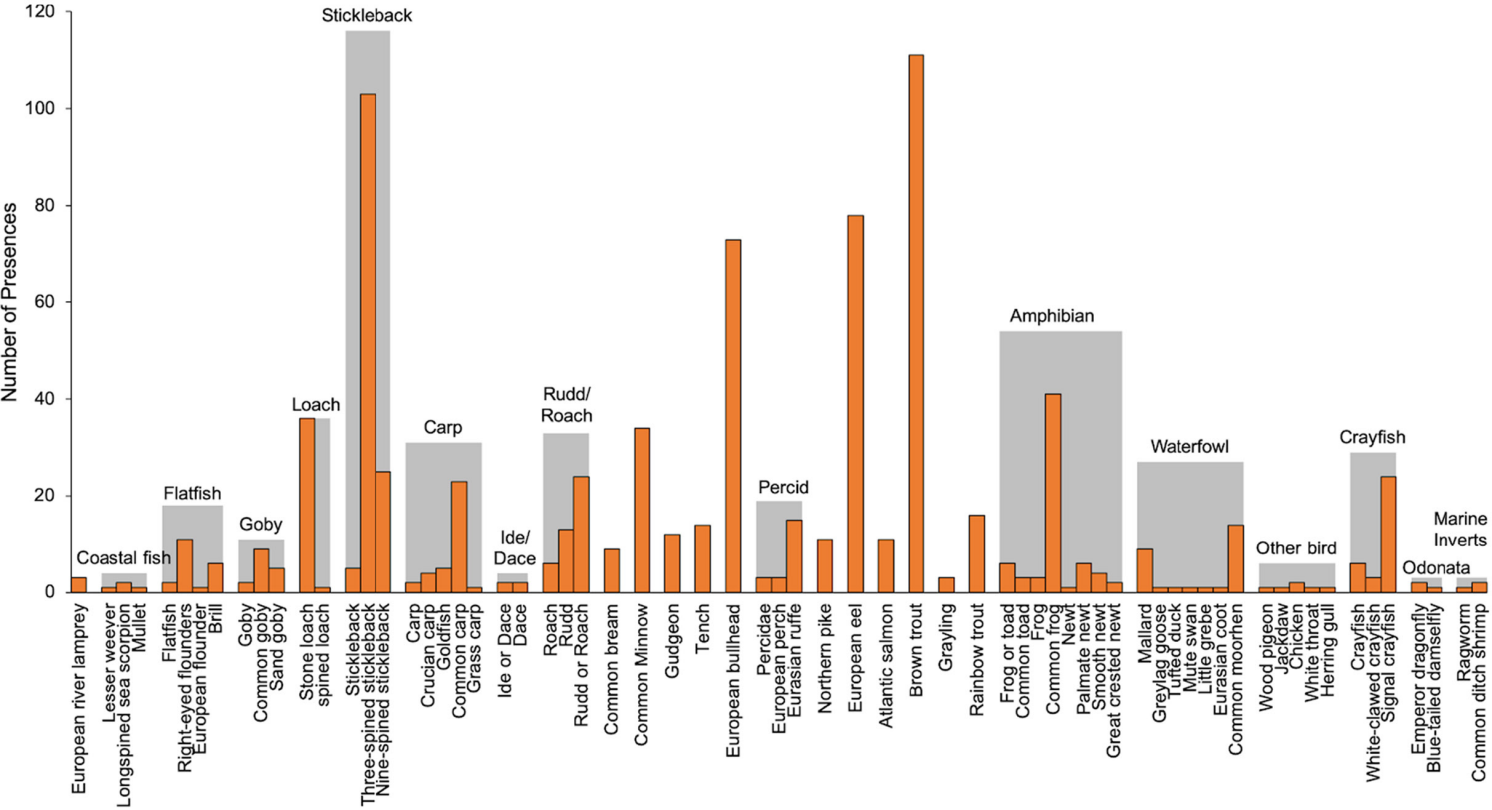
Presence of prey groups (gray) and the taxa that contributed to each prey group (orange) in the diet of Eurasian otters (*Lutra lutra*). Data were obtained by combining identifications made through morphological analysis of prey remains and DNA metabarcoding of feces obtained from dead otters collected from across England and Wales from 2007 to 2016.

Combining data from morphological analysis and metabarcoding increased the number of trophic interactions recovered, therefore, subsequent analyses to assess dietary variation (and investigate hypotheses iii–vi) were carried out on a combined data set. Following aggregation of taxa into prey groups and removal of groups with less than three presences, data input consisted of 765 occurrences of prey from 26 groups (Figure 2) across 268 otters, with a mean of 2.85 prey groups per otter. The most frequent prey groups in the diet of otters were stickleback (39%), brown trout (37%), eel (26%), and bullhead (24%). Model‐based ordination showed that most prey groups cluster close together, suggesting most otters have a similar dietary composition (Figure S7); although, marine and coastal prey (“coastal fish,” “marine inverts,” “flatfish,” and “goby”) appeared to cluster closer together in both ordinations and Cyprinidae (“roach/rudd,” “ide/dace,” “carp,” and bream) clustered together.

**FIGURE 3:**
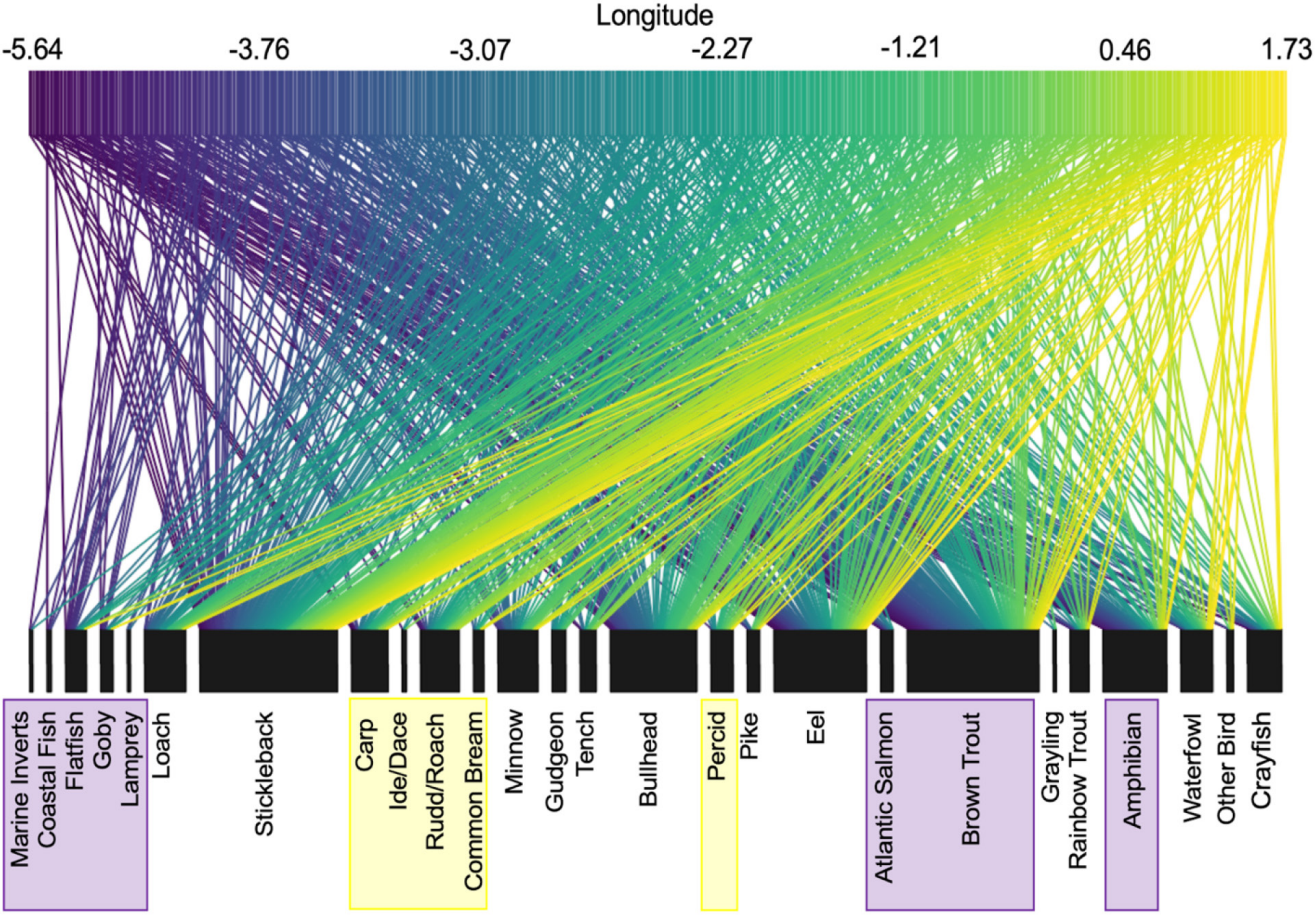
Frequency of occurrence of prey items in the diet of Eurasian otters (*Lutra lutra*) at different longitudes. Data were obtained by combining identifications made through morphological analysis of prey remains and DNA metabarcoding of feces collected from dead otters across England and Wales between 2007 and 2016. The width of the lower boxes is proportional to the frequency of occurrence of each taxon in the diet of otters and the width of each line connecting the upper and lower boxes is proportional to the number of otters from a particular longitude that consumed that prey item. Prey groups highlighted by colored boxes represent those with greater frequencies in western regions (purple) or in eastern regions (yellow).

**FIGURE 4:**
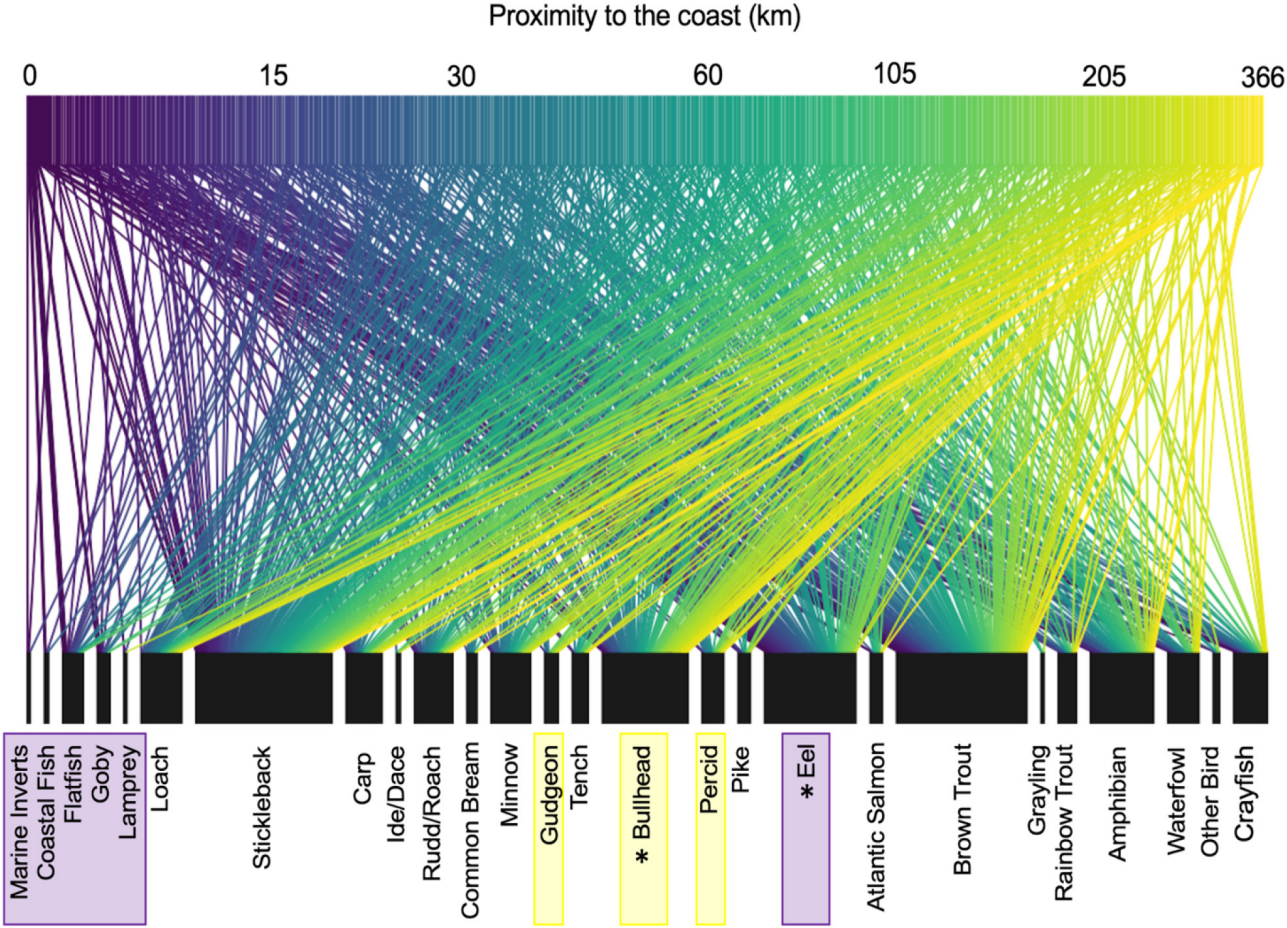
Frequency of occurrence of prey items in the diet of Eurasian otters (*Lutra lutra*) at different coastal proximities. Data were obtained by combining identifications made through morphological analysis of prey remains and DNA metabarcoding of feces collected from dead otters across England and Wales between 2007 and 2016. The width of the lower boxes is proportional to the frequency of occurrence of each taxon in the diet of otters and the width of each line connecting the upper and lower boxes is proportional to the number of otters from a particular distance from the coast that consumed that prey item. Prey groups highlighted by colored boxes represent those with greater frequencies near the coast (purple) or inland (yellow), and * shows specific prey groups that were significantly associated with proximity to the coast.

At the community level (i.e., changes in overall composition of otter diet rather than prey‐specific associations), distinct otter diets were significantly associated with longitude (MGLM: LRT Deviance = 69.73, *p* = .001) and distance from the coast (MGLM: LRT Deviance = 78.52, *p* = .001). Most prey species were observed at all longitudes and all distances from the coast; however, subtle changes in occurrences of certain species drove changes in the composition of otter diets across these variables. Longitudinal variation appeared to be primarily driven by greater frequencies of occurrence for salmonids, amphibians, and marine/estuarine prey in the west, with more cyprinids and percids occurring in the east (Figure 3). Coastal proximity variation was primarily driven by greater occurrences of marine/estuarine prey and eels near the coast and bullhead occurring more inland (Figure 4).

Prey‐specific associations were only found for distance from the coast; eels were consumed significantly less with increasing distance from the coast (MGLM: LRT Deviance = 15.54, *p* = .005; Figure S8), whilst bullhead consumption significantly increased with distance from the coast (MGLM: LRT Deviance = 12.22, *p* = .026; Figure S8). No specific prey was associated with longitude and no significant associations were found between the dietary variation of otters and sex, length, body condition, the proportion of urban land‐use, water habitat type, season, or year.

## 4 DISCUSSION

Otters expressed high dietary plasticity across broad spatiotemporal gradients and abiotic variables, likely reflecting the opportunistic foraging behavior of otters. Variation across landscapes and time likely reflects the distribution of otter prey across different habitats, at least for those prey preferentially predated by otters (Boyer et al., 2015; Deiner et al., 2017; Hawlitschek et al., 2018). The broad range of prey identified in the diet of otters across England and Wales thus likely reflects their generalist foraging behavior and ability to take prey from a range of habitats.

### 4.1 Comparison of methods

Previous studies comparing morphological and molecular analysis of predator diet using feces suggest the two methods detect similar prey items at similar relative frequencies (Casper et al., 2007; Hope et al., 2014; Jeanniard‐Du‐Dot et al., 2017; Thalinger et al., 2016). In comparison, studies using otter feces suggest that while the species identified by each method overlap, the relative frequencies differ (Marcolin et al., 2020; Pertoldi et al., 2021). Our findings using feces obtained from the guts of otter carcasses align with comparison studies using otter feces, suggesting that while there were similarities in the prey identified through morphological analysis and DNA metabarcoding, the relative frequencies at which these prey were detected differed between the two methods (Figure 1 data). The dietary compositions determined by the two methods differed between methods often more than the variation between samples (Figure S6), highlighting the differences in prey detections and thus the complementarity of these approaches. The findings of this study extend beyond previous comparisons using otter feces by using multiple metabarcoding markers (i.e., COI and 16S), facilitating a greater range of prey detections and thus allowing us to compare traditional methods (i.e., morphological identification) against a more comprehensive metabarcoding dataset.

Our findings showed DNA metabarcoding detected a greater range and frequency of prey, and to a greater taxonomic resolution, than morphological analysis of prey remains. Metabarcoding detected easily digested prey (e.g., European river lamprey) and more presence of typically larger fish that may have only been partially consumed (e.g., brown trout, *Salmo trutta*). Although rare, some prey presences in a few individuals were only detected through morphological analysis, possibly due to differential gut DNA retention times (Carss & Parkinson, 1996) resulting in prey hard remains surviving longer than DNA (Casper et al., 2007; Tollit et al., 2009). Morphological analysis underestimated frequently consumed prey (e.g., brown trout) and attributed a large proportion of the diet to lower frequency prey (e.g., loach), reflecting a finding by Lanszki et al. (2015) that less prevalent food types are more frequently morphologically identified in feces due to differential gut retention times of prey remains (Carss & Parkinson, 1996; Carss & Nelson 1998). When rerunning the model used in this study with just molecular or morphological data alone (Appendix S3), the ecological conclusions change markedly, with the combined approach representing variation across both data sets. The choice of method thus impacts the ecological conclusions made from these data. The disparate ecological conclusions reached by using each data set alone highlights the risk in basing ecological analyses and management decisions on single‐method studies but also the strength in combining approaches. Although many prey were more likely to be detected using metabarcoding, both data sets contained unique detections, highlighting their complementarity (Figure S6). Molecular and traditional techniques can be very effectively merged for a more complete trophic network construction (Cuff, Windsor, et al., 2022) and, in this instance, a combined approach gave a more comprehensive description of the otter diet.

### 4.2 Dietary composition

Otters primarily predated freshwater fish, with the most frequently consumed prey identified as stickleback, brown trout, eel, and European bullhead (*Cottus gobio*). When freshwater fish are less available, otters switch to alternative prey (e.g., Almeida et al., 2012; Britton et al., 2006; Krawczyk et al., 2016; Remonti et al., 2010), as also exhibited by other generalist predators (e.g., Rosenblatt et al., 2015; Spencer et al., 2017; Tobajas et al., 2016; Xu et al., 2012; Yeager et al., 2014). In the current study, amphibians (predominantly common frog, *Rana temporaria*) were the most frequent nonfish prey consumed, followed by waterfowl (predominantly common moorhen, *Gallinula chloropus*) and crayfish (predominantly the invasive signal crayfish, *Pacifastacus leniusculus*). Consumption of signal crayfish and grass carp, *Ctenopharyngodon idella*, highlights an ecosystem service provided by otters through the biological control of abundant invasive freshwater species. These results largely align with previous studies, suggesting that the composition of otter diet in Britain may reflect prey abundances (i.e., density‐dependent predation), with otters more likely to consume the most abundant species available (Almeida et al., 2012; Copp & Roche, 2003; Miranda et al., 2008).

Protected species (e.g., great crested newt, *Triturus cristatus*, white‐clawed crayfish, *Austropotamobius pallipes*; Stroud, 2017) are typically less available due to their rarity, and otters more frequently took comparatively common species. Protected species only comprised a small proportion of otter diet in this study, suggesting these are rare predation events and are unlikely to significantly impact protected species. An exception to this is the European eel, a critically endangered species with a declining population (Aprahamian & Walker, 2008; Bark et al., 2007; ICES, 2019). Eels have long been reported as a favored prey of otters (Britton et al., 2006; Copp & Roche, 2003; Miranda et al., 2008), but studies have found as eel populations decline so does predation by otters (Almeida et al., 2012; Copp & Roche, 2003; Kruuk, 2014; Moorhouse‐Gann et al., 2020). Here we found otters are still frequently consuming eels despite their decline. This disparity between studies suggests further research into otter‐eel dynamics and the threats otters may present to future eel recruitment is required. Otters also consumed species stocked by fish farms (e.g., carp and rainbow trout, *Oncorhynchus mykiss*), which is a concern for anglers and aquaculture management, as well as a source of risk for otters given their conflict with these parties (Grant & Harrington, 2015; Poledníková et al., 2013; Vaclavikova et al., 2011). Stocked fish were not found in the majority of otter diets though, with otters more likely to consume wild counterparts, particularly smaller bodied fish such as bullhead as reported in other studies (Britton et al., 2006; Grant & Harrington, 2015; Lanszki et al., 2015; Lyach & Čech, 2017).

### 4.3 Spatial variation

Greater frequencies of marine prey were observed in the diet of otters closer to the coast, reflecting the tendency of otters to opportunistically consume prey from different habitats (Beja, 1991; Clavero et al., 2004; Jędrzejewska et al., 2001; Krawczyk et al., 2016; Reid et al., 2013). Otters utilize marine prey to different extents, with individuals in the Scottish Isles specializing in marine prey (e.g., Kruuk & Moorhouse, 1990; Watt, 1995) while coastal otters in mainland Britain and Europe consume marine prey less frequently (Beja, 1991; Clavero et al., 2004; Heggberget & Moseid, 1994; Moorhouse‐Gann et al., 2020; Parry et al., 2011). In this study, consumption of marine prey only constituted a small proportion of the diet, thus implying that most otters in England and Wales exploit marine species infrequently or not at all. As otter populations recover and expand their distribution, it is possible that exploitation of marine habitats will increase, either due to increased competition for freshwater prey or as coastal individuals gain experience hunting marine prey. Proximity to the coast was also associated with prevalence in the diet of two of the most dominant prey: consumption of eel declined and bullhead increased inland. While bullhead is abundant in a variety of habitats (both upland and lowland; Tomlinson & Perrow, 2003), eel abundances tend to decline with increasing distances from the tidal limit (Ibbotson et al., 2002), leading to otters switching prey as bullhead become more available than eels. Previous studies suggest otters switch from eel to common species, such as bullhead and trout, as eel populations decline (e.g., Almeida et al., 2012; Moorhouse‐Gann et al., 2020); however, our observations suggest that despite declines, eel were still taken more frequently than bullhead between 2007 and 2016.

Variation in otter diet with longitude reflected changing prey distributions, with Salmonidae consumed more frequently in the west, and Cyprinidae and Percidae in the east, consistent with population densities of these families (e.g., Common carp, *Cyprinus carpio*: NBN atlas, 2020a; European perch, *Perca fluviatilis*: NBN atlas, 2020b; Atlantic salmon, *Salmo salar*: NBN atlas, 2020c). These findings reflect the opportunistic foraging behavior of otters, with individuals more likely to encounter and consume abundant prey and support a finding by Harper et al. (2020) that variation in prey availability over fine spatial scales can drive dietary differences in otters. We also observed more amphibian and marine species being consumed by western otters, potentially suggesting a greater reliance on these species as alternative prey, or greater availability in these regions (e.g., increased opportunity to feed on marine prey due to more coastline in western regions). Opportunistic foraging was further implied by the lack of dietary differences between otters from different aquatic habitat types, suggesting that otters are utilizing prey from a variety of habitats within their range, rather than focusing on the nearest habitat. There was also no association between dietary composition and the degree of local urban or rural habitat, suggesting that neither prey availability, nor otter foraging behavior, varies considerably where waterways pass through urban areas.

### 4.4 Temporal variation

Previous otter dietary studies using morphological analysis have found distinct seasonal peaks in amphibian consumption during spring and winter (e.g., Clavero et al., 2005; Moorhouse‐Gann et al., 2020; Parry et al., 2015), with slightly higher frequencies in winter when amphibians are more vulnerable and in spring when they aggregate for breeding (Beebee, 2013). We found broadly similar frequencies across the seasons, potentially reflecting the improved detection of fish species found using metabarcoding and thus altering the relative importance of amphibians during these months. Similarly, the invasive signal crayfish, while consumed, was neither preferentially taken during a particular season nor comprised a large proportion of the diet. In Mediterranean regions, invasive crayfish (primarily red swamp crayfish, *Procambarus clarkii*) are an important dietary element for otters (Adrian & Delibes, 1987; Barrientos et al., 2014; Beja, 1996; Correia, 2001) particularly during droughts when fish are less available. The lack of interaction between British otters and signal crayfish may be due to greater environmental stability in temperate regions, providing otters with the opportunity to frequently consume fish species throughout the year.

We expected the diet of otters to reflect seasonal and annual changes in prey abundance and distribution (Hayhow et al., 2019); however, no significant temporal trends were observed over the 10‐year study. Earlier studies have found fewer eels in the diet of otters in line with eel population declines (Almeida et al., 2012; Copp & Roche, 2003; Kruuk, 2014; Moorhouse‐Gann et al., 2020; respectively reporting years 1991–2000, 1970–2010, 2003–2013, and 1994–2010). The apparent consistency in eel predation shown by the current study may reflect a stabilization in eel populations in later years (2007–2016), although at lower abundances. We also expected to observe greater consumption of invasive species by otters over time as invasive species become more abundant with population increases (e.g., signal crayfish; Holdich et al., 2014; Sibley et al., 2002), yet invasive species comprised only a small proportion of otter diet consistently throughout the study. This may indicate a preference by otters for native species, as observed in Mediterranean otters (Blanco‐Garrido et al., 2008), or lower abundance of invasive compared with native prey. However, as invasive species continue to undergo population expansions and become more available to otters, greater consumption may be observed (Balestrieri et al., 2013).

### 4.5 Biotic variation

Our data suggest that there were no demographic (i.e., sex, size, or body condition) differences in the diet of otters. This contrasts a recent study by Moorhouse‐Gann et al. (2020) which found an association between high‐value prey and the body condition of otters. Although the discrepancy between studies may be due to the shorter time frame or smaller sample size investigated in this study, it may also reflect methodological differences. It is possible that the increased frequency of higher‐quality prey species revealed by metabarcoding reflects the detection of smaller (e.g., juvenile) prey individuals not distinguished morphologically. Although identified as high‐quality species, such prey may represent relatively little nutritionally. While metabarcoding provides a greater insight into the species consumed by a predator, it cannot reveal the size or number of prey consumed (Deagle et al., 2013; Elbrecht & Leese, 2015; Hawlitschek et al., 2018; Mata et al., 2019; Pawluczyk et al., 2015; Piñol et al., 2015), potentially overlooking an important aspect of demographic variation. For example, adult otters might consume primarily large trout, whereas young otters might focus on small fry. Metabarcoding cannot differentiate between the size or number of prey consumed, and although morphological analyses can (Britton et al., 2006; Grant & Harrington, 2015; Lyach & Čech, 2017), this is extremely laborious and relies on particular hard parts being present within a sample (e.g., fish vertebrae used to estimate size), which may be misleading where, for example, otters have only consumed the flesh of prey and not hard parts, or only part of an animal (Adámek et al., 2003; Kortan et al., 2007; Ruiz‐Olmo et al., 1998). These findings demonstrate how comparing and combining these complementary methods of dietary analysis can more clearly identify the prey consumed by otters of different demographic groups compared with assessing such dietary variation using morphological or metabarcoding analyses in isolation.

### 4.6 Limitations

Using samples collected from dead otters allowed us to collect data over a broad spatiotemporal scale without using invasive methods and limited the influence environmental variables (e.g., UV radiation and external contamination subjected to feces collected from visual surveys) may have had. However, DNA degradation may have occurred before an otter carcass was collected, potentially impacting metabarcoding data. To limit the influence of sample degradation, we only used otters classified as “not very degraded.” Additionally, read counts from metabarcoding data can be influenced by biases introduced during the sample collection (e.g., DNA degradation) and processing (e.g., primer bias), resulting in the contentious debate regarding the utility of read counts for approximating prey abundance data (Deagle et al., 2013, 2019; Murray et al., 2011; Piñol et al., 2018; Thomas et al., 2016). We therefore did not use read counts under the assumption that they cannot be used to infer reliable prey quantities in diet and instead limited this study to presence‐absence data. While observational and morphological analyses are frequently used to infer quantities of prey consumed, this study did not acquire this data due to the difficulty involved in ascribing whole‐organism counts to fragmented hard parts, ultimately resulting in data more directly comparable with the molecular data. Increasingly, metabarcoding studies are using technical replicates (e.g., triplicates) to identify errors introduced during sample processing and increase accuracy (Alberdi et al., 2018). Replicating metabarcoding workflows is, however, time‐consuming, laborious and expensive, and sequencing depth has been demonstrated as more important in determining accuracy and detectability in such studies (Singer et al., 2019; Smith & Peay, 2014). Although it was not possible for this study, we would recommend replication PCRs for other studies intending to perform similar analyses.

Given the difficulty associated with accurate morphological identification of prey remains from fecal matter, and similar issues with DNA barcodes of closely related prey, some identifications were not resolved to the species level. Equally, the common reliance on metabarcoding on public reference databases can introduce errors resulting from the misidentification of barcoded specimens, the presence of only partial sequences, or the omission of some species altogether (Gerwing et al., 2016; Zinger et al., 2019). Such issues with metabarcoding will likely be alleviated by ongoing initiatives to comprehensively barcode British fauna and flora (The Darwin Tree of Life, 2020), after which the accuracy of these methods will further improve and fewer misidentifications will be made (Gerwing et al., 2016; Hibert et al., 2013). It is also likely that some occurrences reflect secondary predation (Bowser et al., 2013; Galan et al., 2018; Pompanon et al., 2012; Sheppard et al., 2005), although DNA degradation prior to consumption (Kamenova et al., 2018; Nielsen et al., 2018) and the use of minimum sequence copy thresholds (Drake et al., 2022) likely minimize this potential source of error in metabarcoding data.

## 5 CONCLUSIONS

Metabarcoding provides a methodological advance for the study of generalist apex predator diets, providing greater precision for the identities and frequencies of species consumed compared with traditional morphological methods. Otters exploited a broad range of prey from different habitats, with dietary variation likely reflecting the adaptability of otters to temporal and landscape differences in prey distributions. The dietary plasticity of otters observed here has likely aided the recovery of British populations (Peers et al., 2014; Van Baalen et al., 2001) and may increase the resilience of these populations to future environmental stressors. Greater dietary resolution also provided an insight into prey population dynamics within the environment, supporting the use of metabarcoding studies of generalist predators to help guide biodiversity management, especially where surveying may be difficult (Boyer et al., 2015; Deiner et al., 2017; Deiner & Altermatt, 2014; Hawlitschek et al., 2018). These findings provide a robust framework for future dietary assessments of otter populations across large spatiotemporal scales but also valuable insights into their foraging ecology with important implications for the population dynamics of this recovering apex predator.

## AUTHOR CONTRIBUTIONS

**Lorna E. Drake:** Conceptualization (lead); data curation (lead); formal analysis (lead); investigation (equal); methodology (lead); project administration (lead); visualization (lead); writing – original draft (lead); writing – review and editing (equal). **Jordan P. Cuff:** Data curation (equal); formal analysis (equal); investigation (equal); visualization (equal); writing – review and editing (equal). **Sergio Bedmar:** Data curation (equal); investigation (equal); methodology (equal); writing – review and editing (equal). **Robbie McDonald:** Conceptualization (equal); funding acquisition (equal); investigation (equal); project administration (equal); supervision (equal); writing – review and editing (equal). **William O. C. Symondson:** Conceptualization (equal); funding acquisition (equal); investigation (equal); project administration (equal); supervision (equal); writing – review and editing (equal). **Elizabeth A. Chadwick:** Conceptualization (equal); funding acquisition (equal); investigation (equal); project administration (equal); supervision (lead); writing – review and editing (equal).

## CONFLICT OF INTEREST STATEMENT

The authors have no competing interests to declare.

## Supporting information


Appendix S1

## Data Availability

Data are available at dryad under two sources. Metabarcoding data, including raw sequence data and data that has been filtered following application of bioinformatic and post‐bioinformatic thresholds, are available at Drake and Cuff (2021). Morphological data alongside Metabarcoding data filtered for dietary analyses can be found at Drake et al. (2023).
