# Supplementary material for: Otterly delicious: Spatiotemporal variation in the diet of a recovering population of Eurasian otters (Lutra lutra) revealed through DNA metabarcoding and morphological analysis of prey remains
Source: Ecol Evol. 2023 May 10;13(5):e10038. doi: 10.1002/ece3.10038 (PMC10170393; doi:10.1002/ece3.10038)
Supplement: Supplementary file 1 — Appendix S1 [file ECE3-13-e10038-s001.docx]

## Appendix

**Appendix S1: High Throughput Sequencing**

**S1.1 Sample processing**

Samples were obtained from the Cardiff University Otter Project collection, a national monitoring programme for dead otters sampled from across Great Britain (https://www.cardiff.ac.uk/otter-project). Most otters collected were killed by road traffic accidents, with a minority dying through drowning, being shot, starvation, or disease. Information on date (year and month) and location (as grid reference) of carcass collection were recorded at the site of collection. Grid references were used to plot data for spatial analysis. Detailed post-mortems were performed for each carcass (data not presented) during which faecal samples were collected, wrapped in foil and stored at -20 °C. Faecal samples were thawed and homogenised by hand in zip lock bags, and three sub-samples of ~200 mg each were taken and stored in a 2 ml Eppendorf tube at -80^o^C. DNA extraction was carried out in a laminar flow using the QIAamp DNA Mini Stool Kit following the manufacturer’s protocol (Qiagen, Hilden, Germany), except with only half the recommended volume of buffers and InhibitEX tablets used (Deagle et al. 2005). Extraction negatives were included alongside each group of extractions to check for contamination (King et al. 2008) and successfully extracted DNA was stored at -20 °C.

**S1.2 DNA amplification**

Primers underwent *in silico* testing using ecoPCR (Boyer et al. 2016) and were further tested *in vitro*. Temperature gradient polymerase chain reactions (PCRs) were performed to ascertain the optimal annealing temperature which would amplify most of the prey taxa but reduce the amount of predator DNA amplified. PCRs were run using 5 µl reaction volumes under the following conditions: 2.5 µl multiplex (Qiagen), 1.75 µl RNA/DNA free water, 0.05 µl BSA (0.05ug/ml), 0.1 µl of each primer at 10 µm concentration and 0.5 µl of template DNA, with an initial denaturation at 95 °C for 15 minutes, 35 cycles of 94 °C for 30 seconds, annealing temperature (40-60 °C, temperature gradient dependent) for 90 seconds, 72 °C for 90 seconds and a final extension at 72 °C for 10 minutes before being held at 15 °C. Negative (RNA/DNA free water) and positive controls (predator and prey DNA) were included in each PCR. Successful amplifications were visualised via gel electrophoresis using 2 % agarose gel stained with SybrSAFE. In vitro tests showed primer sets amplified desired taxa using an optimal annealing temperature of 57 °C for 16S and 54 °C for COI.

Faecal DNA was amplified using NGS-grade primers with multiplex identifier sequences (or molecular identification tags; MID tags) to enable individual sample identification during bioinformatics analysis. A selection of 16 samples were amplified twice but with different MID-tag combinations to assess PCR errors, contamination and biases introduced by MID-tags. A 25 µl reaction volume was used for MID-tag PCRs under the following conditions; 12.5 µl multiplex (Qiagen), 6.75 µl RNA/DNA free water, 0.25 µl BSA (0.05 µg/ml), 0.5 µl of forward primer at 10 µm concentration, 2.5 µl of reverse primer at 2 µm and 2.5 µl of template DNA using the same PCR cycle as described above but with the optimal temperature for the MID-tagged primers used. Optimal temperatures were identified by constructing temperature gradient PCRs using a selection of MID-tag primer combinations with different annealing temperatures; this revealed the optimal annealing temperatures were 57 °C and 54 °C for 16S and COI MID-tagged primers, respectively. Results from PCRs using MID-tagged primers were visualised using 3.5 µl of product on a QIAxcel (Qiagen), which identifies and visualises concentrations of amplified DNA for each fragment size present. Samples successfully amplified during PCRs using MID-tagged primers (along with necessary negative controls and mock communities) were pooled to create equimolar mixtures; faecal samples and mock communities were pooled relative to their concentration depicted by the Qiaxcel, whilst negatives were pooled using the average concentration for reactions within their specific PCR run. Samples were first pooled by PCR run, giving six pools for 16S and seven for COI tagged samples.

**S1.3 High-Throughput Sequencing (HTS)**

These pools were cleaned and concentrated using a left-side SPRI bead size selection, following the manufacturer's protocol with a SPRI bead ratio of 1.2x for 16S pools and 0.9x for COI pools (Beckman Coulter, Brea, USA). Pool concentrations were determined using Qubit dsDNA high sensitivity assay kit (Thermo Fisher Scientific, Waltham, U.S.A), quality checked using TapeStation 2200 (Agilent, Santa Clara, USA) and combined to create one final equimolar mixture per barcoding region. These final pools underwent library preparation following the protocol outlined in the NEXTflex Rapid DNA-seq kit (Bioo Scientific, Austin, USA) and high-throughput sequencing was conducted on an Illumina MiSeq at Cardiff University Genomics Hub. Due to differing amplicon sizes between the 16S and COI amplicons, the two libraries were sequenced on separate runs using V2 sequencing chip with 2x250bp paired-end reads (expected capacity 12 – 15 million reads; Illumina 2020).

**S1.4 Sequence analysis**

Bioinformatic analyses were carried out using a custom pipeline. Sequences were first checked for truncation of MID tags by determining the proportion of sequence files containing exactly 10 bp before their respective primer. In all cases, the degree of truncation was deemed acceptable (≤10 %). FastP (Chen et al., 2018) was used to check the quality of reads, discard poor quality reads (<Q30, <125 bp long or too many unqualified bases, denoted by ‘N’) and merge read pairs from MiSeq files (R1 and R2). Merged reads were assigned a sample ID based on the MID tags associated with each primer using the ‘trim.seqs’ function of Mothur (Schloss et al., 2009); this also removed the MID tag and primer sequences from the reads. Using the files created by Mothur, reads were demultiplexed to obtain one file per sample ID. Read headers were modified for each file to include the sample ID and reads were then concatenated back into one file. Sequences were denoised (removal of PCR and sequencing errors), clustered into amplicon sequence variants (ASVs) and an ASV table was created using the commands ‘fastx_uniques’, ‘unoise3’ and ‘otutab’ in Usearch (v.11; Edgar, 2016; Edgar, 2020). Taxonomic assignment for each ASV was obtained using the ‘blastn’ command in BLAST+, using a threshold of 97% similarity and e-value of 0.00001, against a downloaded database of DNA barcoding sequences submitted to online databases (e.g., GenBank; Camacho et al., 2009; National Center for Biotechnology Information, 2008). Before assigning taxonomic identities to each ASV, BLAST results were filtered using the dplyr package in R (version 3.6.0) using R Studio (version 1.2.1335; R Core Team, 2019). This was used to retain only accession codes with the top BIT score for each ASV. These data were then processed via MEGAN (version 6.12.3; Huson et al., 2016) to assign taxonomic names to each ASV. As erroneous entries on online databases can prevent species-level assignments, ASVs for which the top BLAST hit (i.e., top BIT score) was not resolved to species level were thus manually checked and assigned the most appropriate taxon. Taxonomic identity for each ASV was added to the ASV table produced by Usearch and reads were aggregated by taxonomic identity for each sample in R using the ‘aggregate’ function with a sum base function. ASVs were allocated taxonomic identities to overcome issues such as over-splitting of taxonomic groups, and to facilitate ecological interpretation of the data, particularly regarding identification of artefacts (e.g., identifying marine species in non-coastal otters).

**S1.5 Minimum sequence copy thresholds**

Filtering methods were enacted in excel using IF formulae. If the read count (i.e., number of reads per sample per taxon) did not pass the designated threshold, then it was converted to zero (rather than subtracting the threshold, thus not altering the remaining read counts). Thresholds were based on artefacts identified in control samples or through taxa unexpectedly occurring in samples, such as taxa from dietary samples in controls, marine taxa associated with otters that did not have access to marine habitats and mock community taxa in negative controls, unused MID tags or dietary samples. Two minimum sequence copy thresholds (MSCTs) were combined as per the recommendation of Drake et al. 2021; a sample-based threshold was used to remove taxa per sample that contributed to less than 0.5 % of a sample’s total reads for 16S and 0.3 % for COI, and maximum contamination was removed by removing reads that were equal to or less than the maximum read count identified in unused MID-tag combinations or negative controls per taxon. Following the use of MSCTs, results were visualised by converting tables of reads into heat charts using the ggplot2 package (Wickham, 2016) in R.


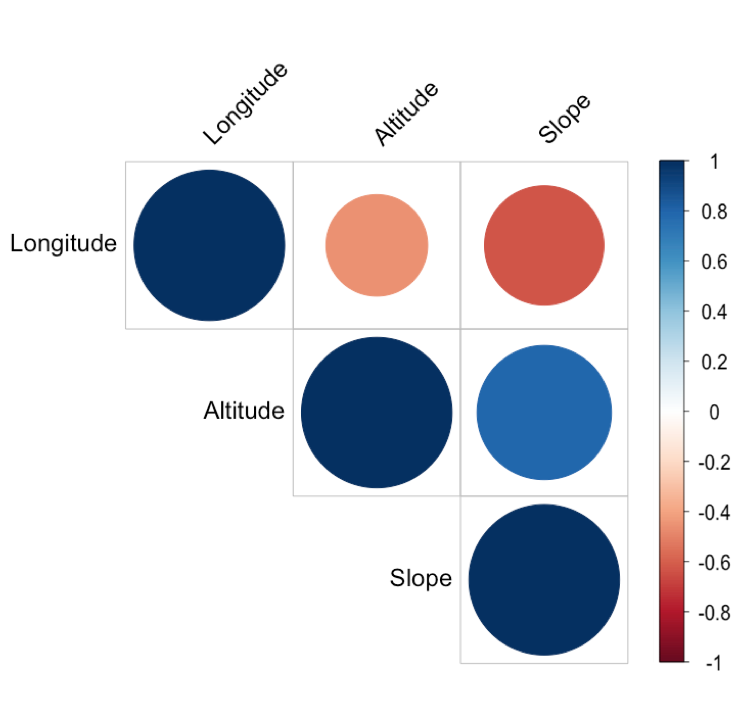


Figure S1. Associations between spatial variables. Plot shows the Pearson’s correlation coefficient. Spatial variables represent the landscape within a 10 km buffer of each individual otter: ‘Longitude’ is the position an otter was found at (center of the buffer) and ‘Altitude’ and ‘Slope are the mean values within each buffer. Blue circles represent positive correlations whilst red circles represent negative correlations. Darker colours and larger circles represent stronger correlations.


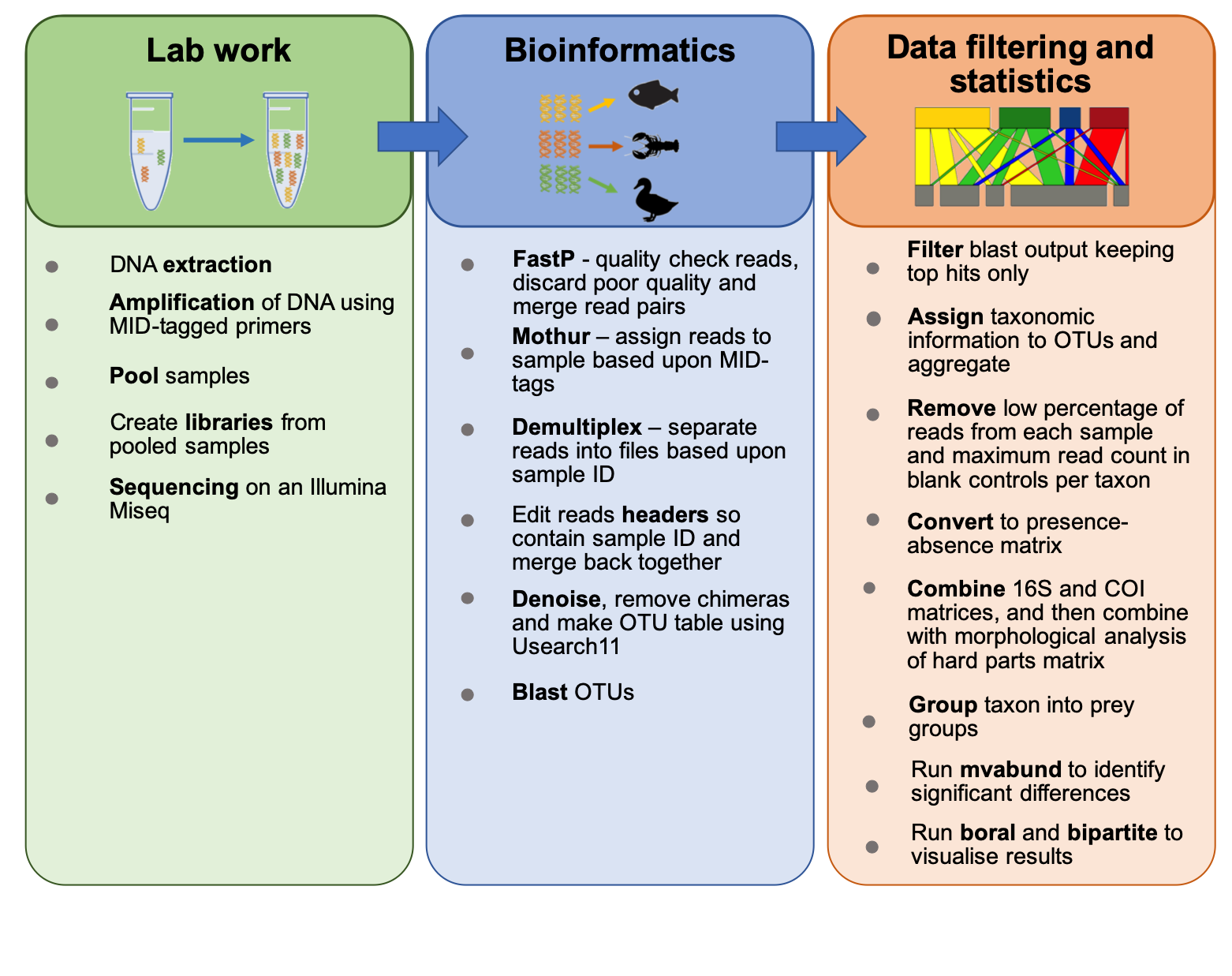


**Figure S2.** Summary of the metabarcoding workflow utilised in this study**.**


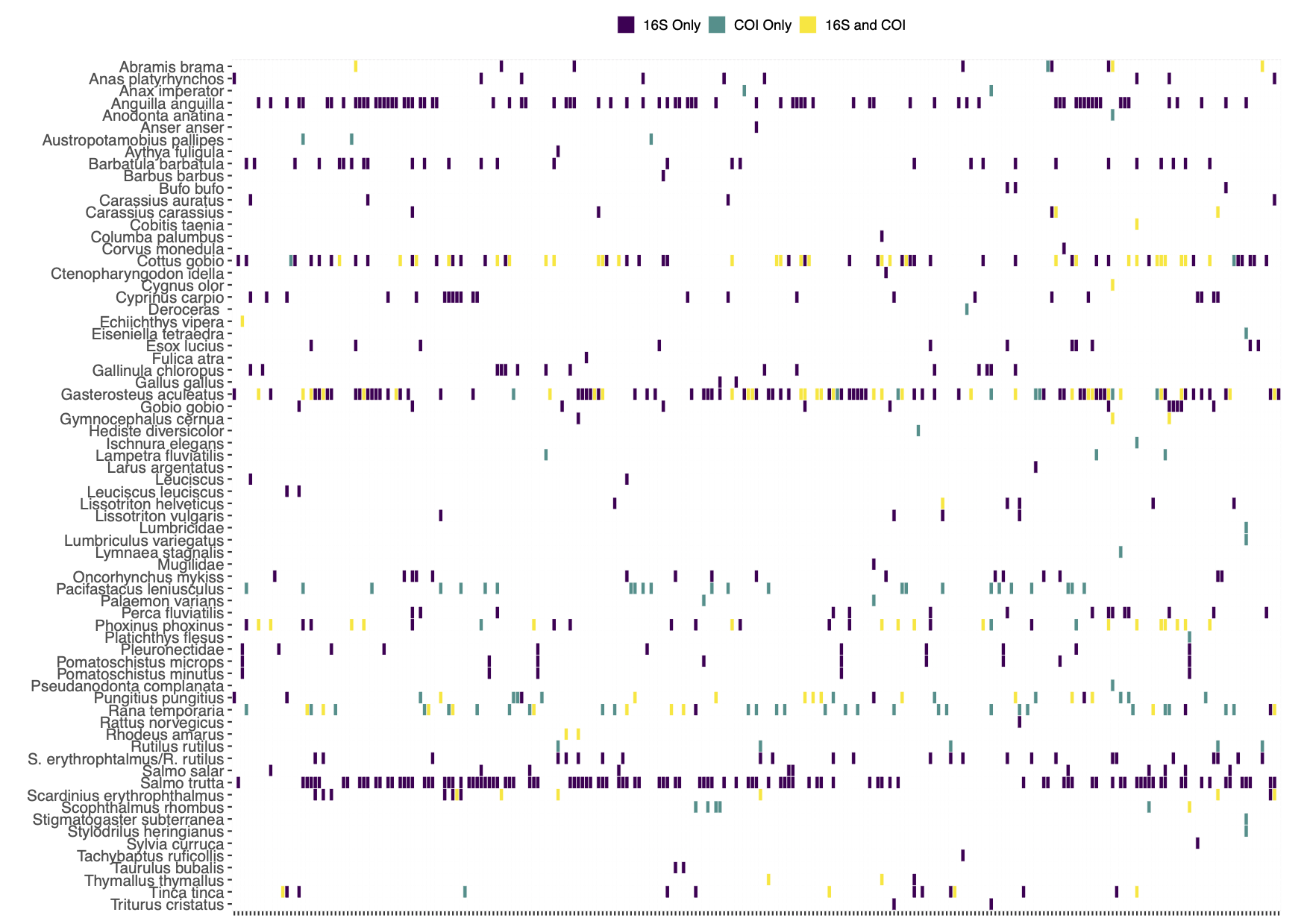


Figure S3. Taxon presences in the diet of Eurasian otters (*Lutra lutra*) using DNA metabarcoding on faecal samples. Purple lines represent presences only identified using the 16S primer set, green presences only identified using the COI primer set, and yellow identified using both primer sets. Each row is a taxon and each column is an individual faecal sample


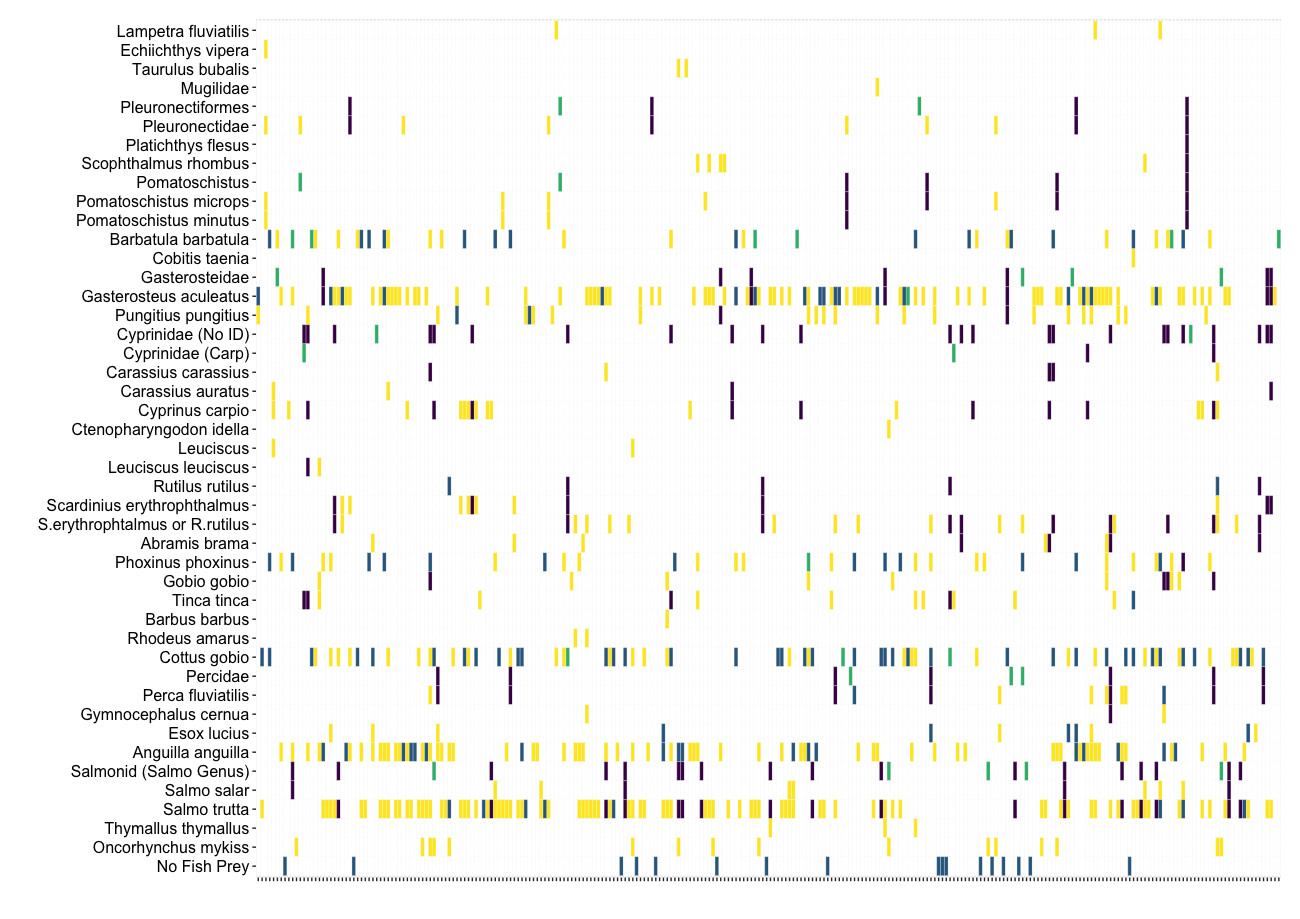


**a)**

Figure S4. Taxon presences in the diet of Eurasian otters (*Lutra lutra*) using morphological analysis of prey remains and DNA metabarcoding on faecal samples for fish taxa (a) and non-fish taxa (b)**.** Colour of squares depicts which method led to the identification: metabarcoding only (yellow), morphological analysis only (green), both at same taxonomic level (blue) and both but at different taxonomic levels (purple). Rows represent taxon and columns represent individual faecal samples. Faecal samples were obtained from dead otters collected from across England and Wales from 2007 to 2016.


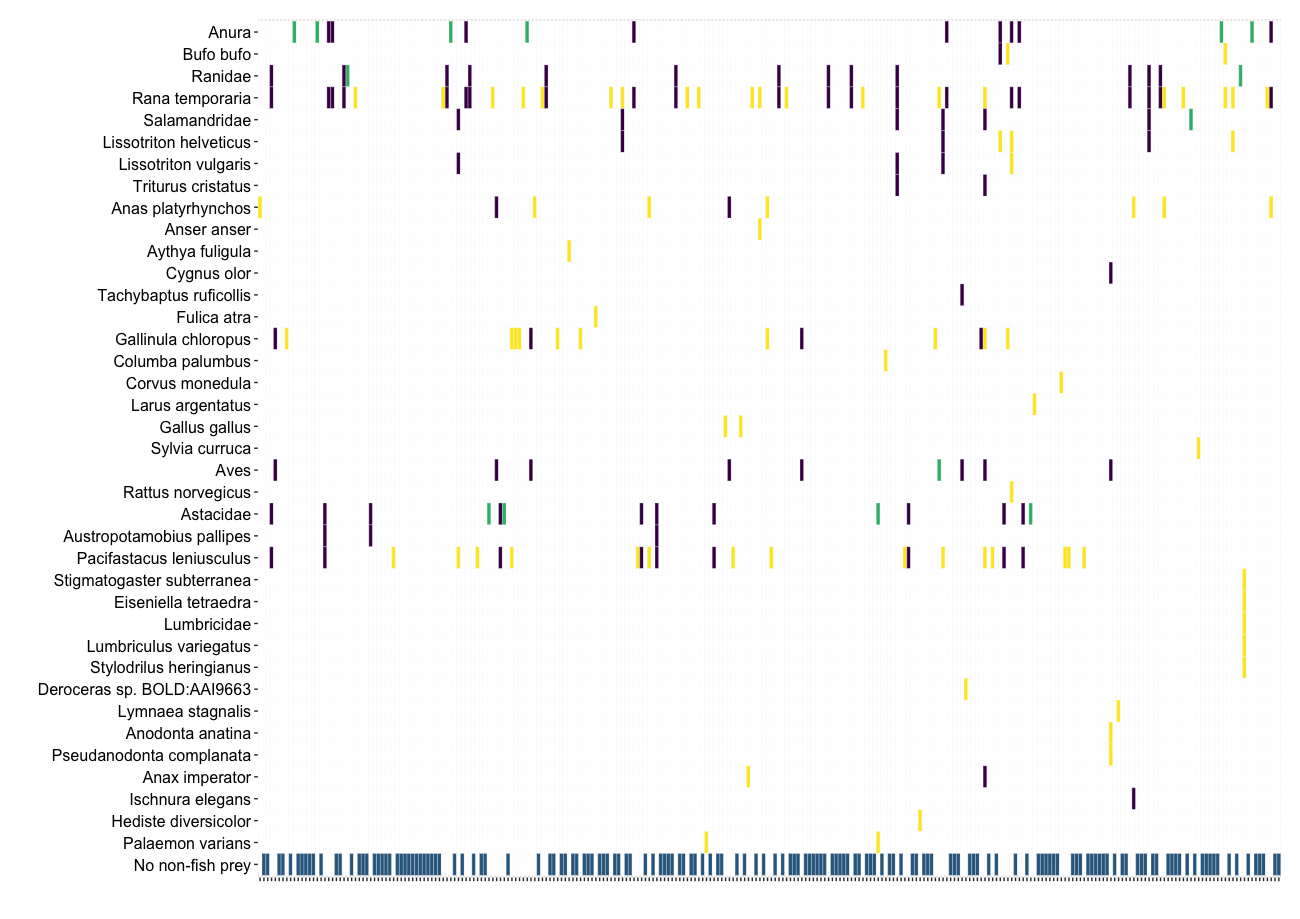


**b)**

**Figure S4.** (continued)


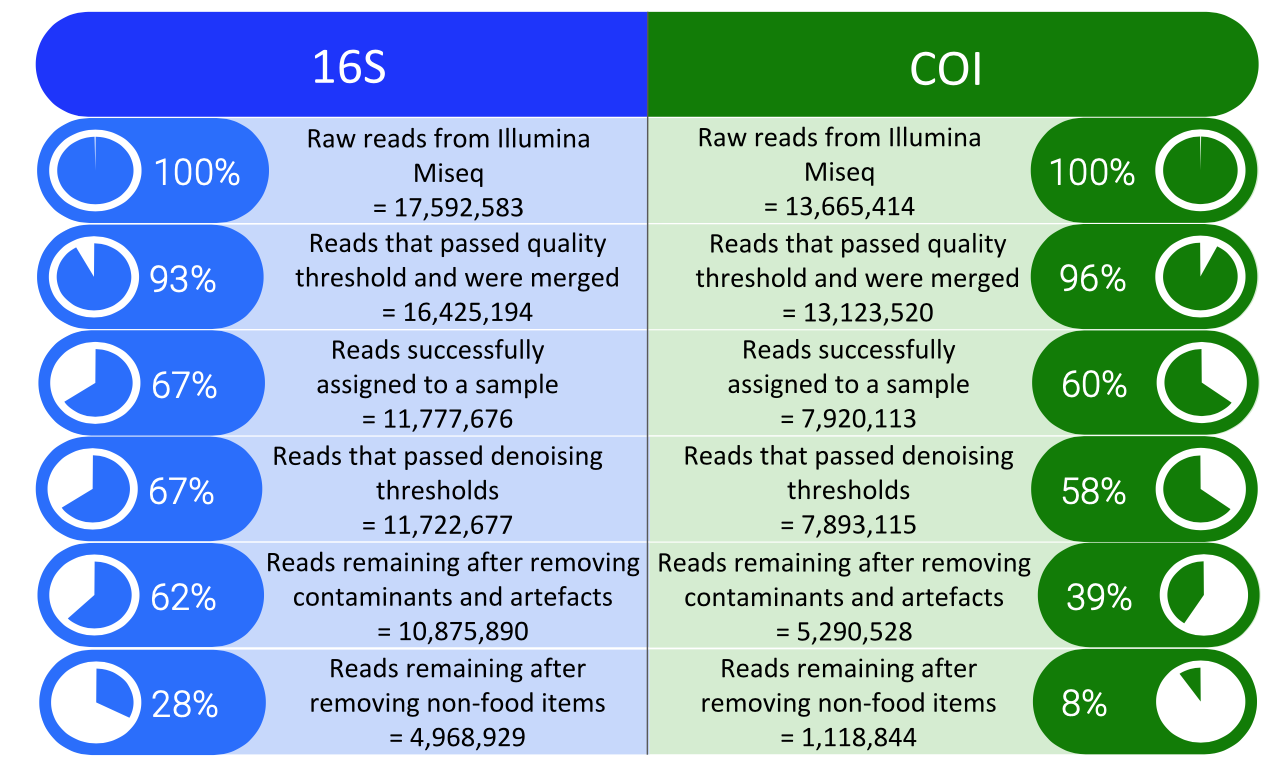


**Figure S5.** Total number of reads remaining in both 16S and COI datasets at each stage of the bioinformatic and data filtering process.


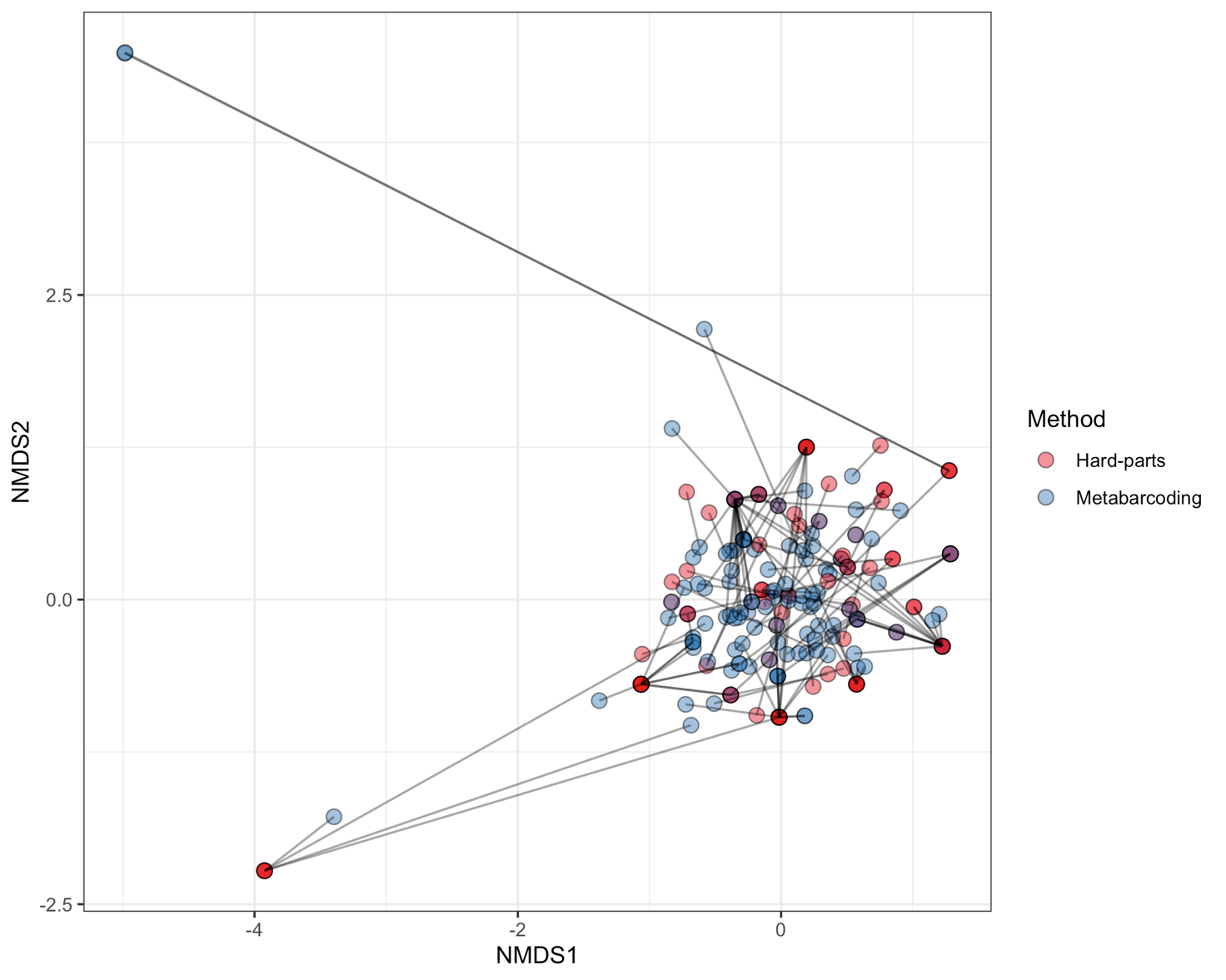


**Figure S6.** Non-metric multi-dimensional scaling showing often higher similarity of dietary data of the same data type (colours; red and blue denoting hard-parts and metabarcoding, respectively) than within samples (points linked by black lines belong to the same sample). Binary matrices for prey detections were combined for the two data types, but each sample represented separately for each method (i.e., not aggregated by sample). Stress = 0.120.


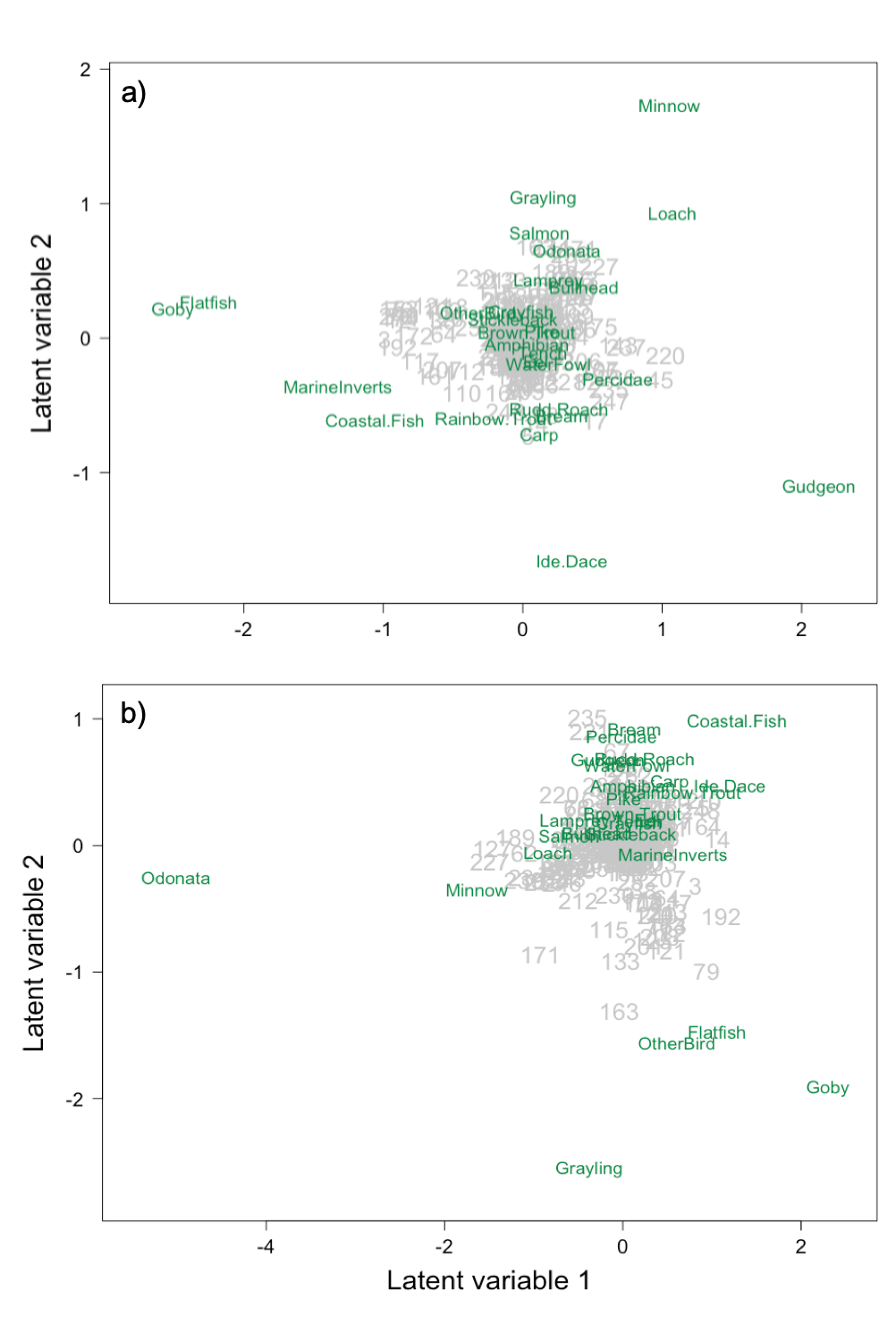


**Figure S7.** Model-based unconstrained residual ordination biplot for Eurasian otter (*Lutra lutra*) diet via boral. Latent variable models using an unconstrained ordination with (a) no predictors and (b) a residual ordination after controlling for the effect of season, coastal proximity and longitude. Each number represents one otter and taxon labels represent prey items; numbers closer together represent otters with more similar diets, and taxon labels closer together represent prey items more likely to co-occur in the diet of otters. In both ordinations most prey items and otters cluster close together, showing no clear pattern in dietary variation. However, in the unconstrained ordination, marine and coastal fish cluster closer to each other and further away from other taxa and Cyprinidae cluster close to one another. Data were obtained by combining identifications from morphological analysis of prey remains and DNA metabarcoding of faeces obtained from dead otters collected across England and Wales between 2007 and 2016.


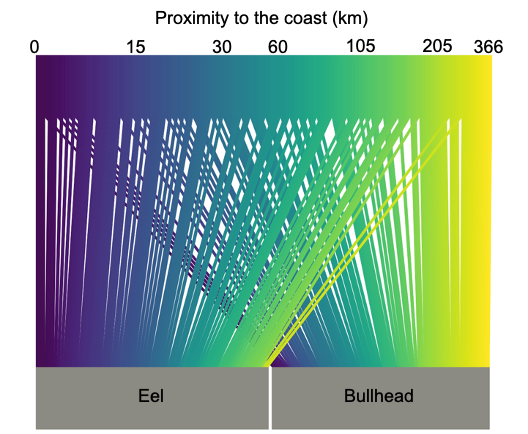


Figure S8. Frequency of occurrence of European eel (*Anguilla anguilla*) and European bullhead (*Cottus gobio*) in the diet of Eurasian otters (*Lutra lutra*) at different coastal proximities. Data were obtained by combining identifications made through morphological analysis of prey remains and DNA metabarcoding of faeces collected from dead otters across England and Wales from 2007 to 2016. The width of the upper boxes and lines connecting upper and lower boxes is proportional to the number of otters at each proximity to the coast that had eel or bullhead, and the width of the lower boxes is proportional to the total frequency of occurrence of eel or bullhead.

**Table S1.** Associated metadata relating to 264 Eurasian otters (*Lutra lutra*) sufficient dietary data was acquired for.


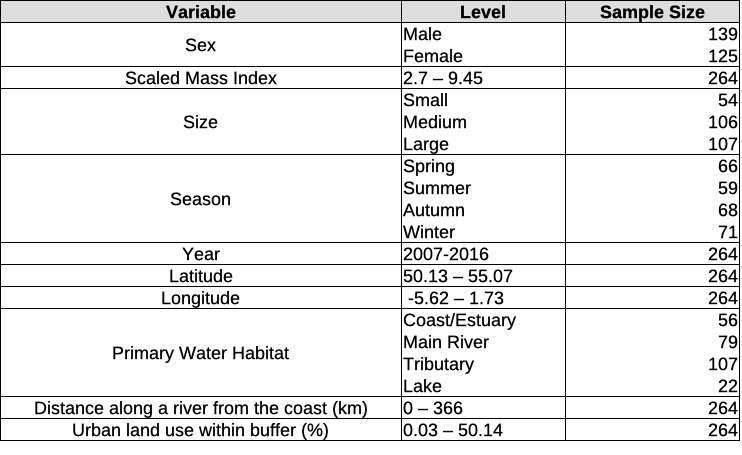


### *Table S2. Taxa obtained from morphological analysis of prey remains in Eurasian otter (Lutra lutra) faecal samples.* ***Latin names and common names are given for each taxon identified, along with whether the taxon was removed before data interpretation and the reason for removal of the taxon.***

Table S3. Taxa obtained from sequencing Eurasian otter (*Lutra lutra*) faecal samples using 16S primers FN2199 (5’- yayaagacgagaagaccct -3’) and R8B7 (5’- ttatccctrgggtarcthgg -3’) (modified from Deagle *et al.* 2009). Latin names and common names are given for each taxon identified following bioinformatic analysis, along with whether the taxon was removed before data interpretation and reason for removal of the taxon. Total read counts and presences per taxa shown were calculated following bioinformatic analysis and artefact removal.

**Table S3.** (continued)

Table S4. Taxa obtained from sequencing Eurasian otter (*Lutra lutra*) faecal samples using COI primers Mod_mCOIintF (5’- ggwacwggwtgaacwgtwtaycc -3’) (modified from Leray *et al.* 2013) and HCO-2198 (5’- taaacttcagggtgaccaaaaaatca -3’) (Folmer *et al.* 1994). Latin names and common names are given for each taxon identified following bioinformatic analysis, along with whether the taxon was removed before data interpretation and the reason for removal of the taxon. Total read counts and presences per taxa shown were calculated following bioinformatic analysis and artefact removal.


**Table S4.** (continued)

**Table S4.** (continued)

**Table S4.** (continued)

**Table S5.** Taxa identified in Eurasian otter (*Lutra lutra*) faecal samples from across England and Wales between 2007- 2016, along with method of identification, the 'prey group' taxa were clustered into and the reason why taxa were grouped. Prey groups removed prior to statistical modelling are indicated by * (poor taxonomic resolution) or ** (total presences <3).


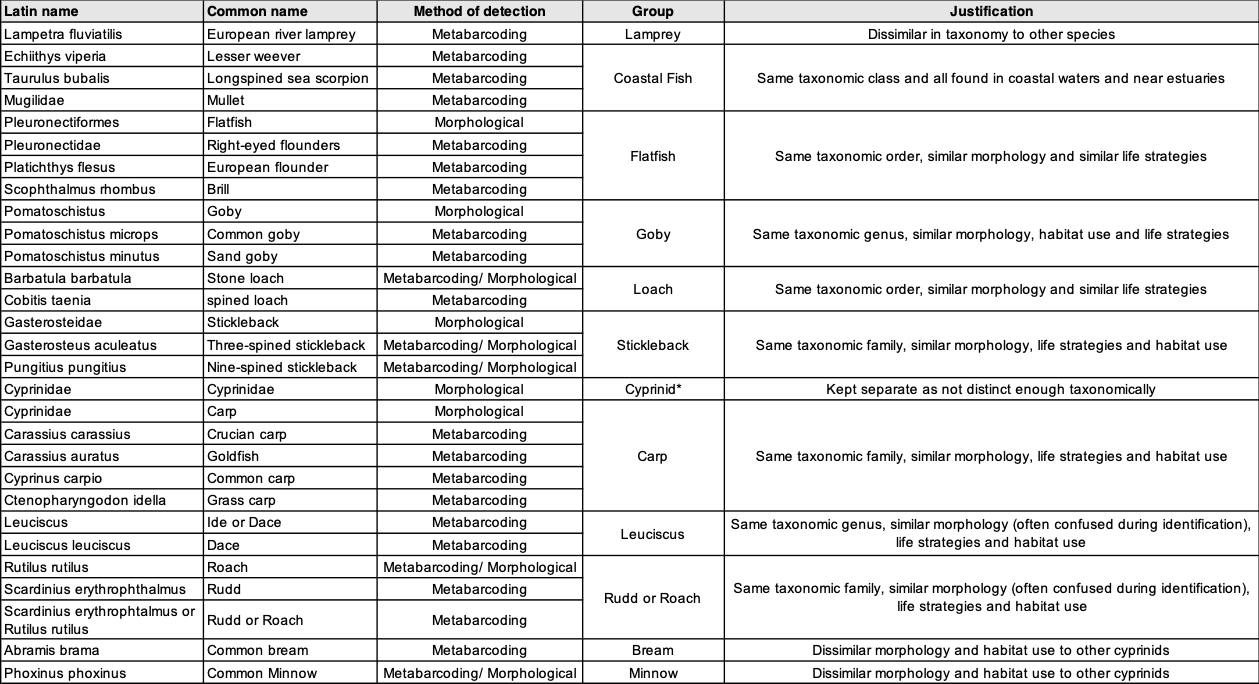


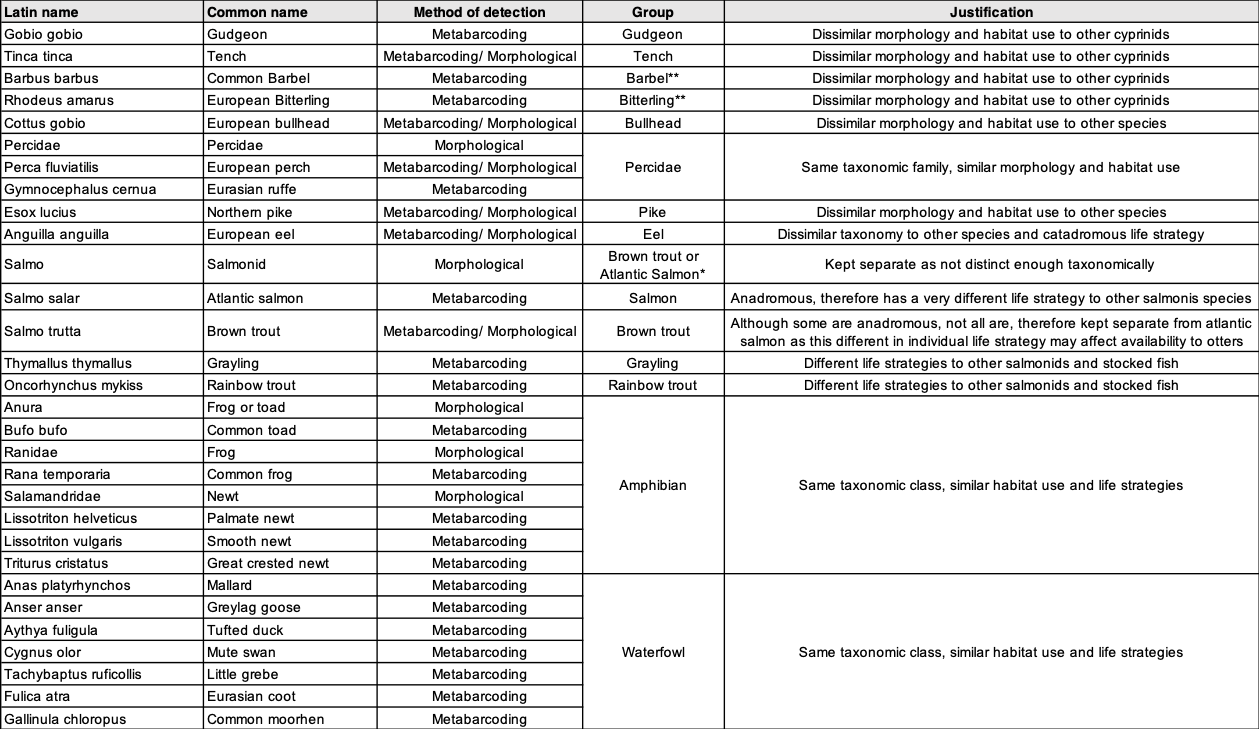


**Table S5.** (continued)


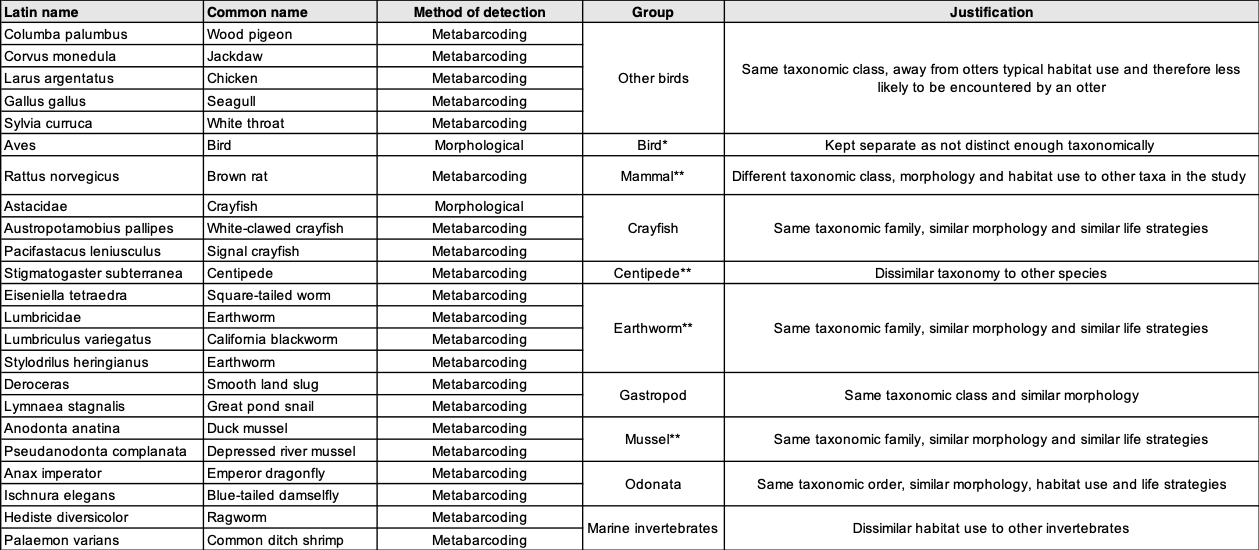


**Appendix S2: Scripts for analysing data in R**

R Code used for analysing metabarcoding data acquired from Eurasian otter faecal samples. Code was run using R [version 3.6.0] and R studio [version 1.2.1335] (R Core Team 2019) and converted into document format using R markdown (Xie *et al.* 2018; Allaire *et al.* 2020). Executable code is presented in grey boxes.

**S2.1 Load packages**

library("corrplot")
library("ggplot2")
library("plyr")
library("reshape2")
library("viridis")
library("scales")
library("RColorBrewer")
library("mvabund")
library("boral")
library ("birpartite")

library("devtools")

library ("vegan")

library ("ggthemes")

**S2.2 Correlation plot**

Load data and extract necessary information

buffer <- read.csv("Otter10kmVariables.csv", header = T)
summary(buffer)
colnames(buffer)
corrcheck <- buffer[ ,c(12,19,13,9,10)]
str(corrcheck)

Check correlation between landscape variables acquired from 10km buffers around each otter

cor1 <- cor(corrcheck)
corrplot(cor1, type = "upper", order = "hclust", tl.srt = 45, tl.col = "black")

**S2.3** **Create heat charts to showing taxonomic identifications by each method**

*Compare metabarcoding 16S and COI identifications*

Load in data and format for plotting

HeatChart <- read.csv("Combined Heat Chart.csv", header = T)
summary(HeatChart)
meltedHeatChart <- melt(HeatChart)

Plot heat chart

ggchart <- ggplot(meltedHeatChart, aes(variable, y = reorder(Taxon, desc(Taxon)))) +
 geom_tile(aes(fill = value), colour = "white") +
 scale_fill_gradientn(na.value = "white", colours=viridis(3),
 values=rescale(c(1,2,3)),
 breaks = c(1,2,3), labels = c("16S Only","COI Only","16S and COI"),
 guide = "legend") +
 coord_fixed(ratio = 3, xlim = NULL, ylim = NULL, expand = TRUE) +
 theme(axis.title.y=element_blank(),
 axis.title.x=element_blank(),
 axis.text.x=element_blank(),
 axis.text.y = element_text(size = 9, colour = "black"),
 legend.position="none")
ggchart

*Compare identifications from morphological analysis of prey remains and metabarcoding*

Load in data and format for plotting

HeatMethods <- read.csv("HTSvsHP_HeatChart.csv", header = T)
HeatMethods$Group <- as.character(HeatMethods$Group)
HeatMethods$Group <- factor(HeatMethods$Group,

levels=unique(HeatMethods$Group))
HeatMethods$Group <- factor(HeatMethods$Group,

levels = rev(levels(HeatMethods$Group)))
summary(HeatMethods)
meltedHeatMethods <- melt(HeatMethods)

Plot heat chart with all data

ggplot(meltedHeatMethods, aes(variable, Group)) +
 geom_tile(stat = "identity", aes(fill = value), colour = "white") +
 scale_fill_gradientn(na.value = "white", colours=viridis(4, direction = -1),
 values=rescale(c(1,2,3,4)),
 breaks = c(1,2,3,4),
 labels = c("Only molecular","Only morphological",
 "Molecular and morphological at same taxonomic level",
 "Molecular and morphological at different taxonomic
 level"), guide = "legend") +
 theme(legend.position="none",
 axis.title.x = element_blank(),
 axis.title.y = element_blank(),
 axis.text.x = element_blank(),
 axis.text.y = element_text(size=10, colour = "black"),
 plot.margin=unit(c(0.5,0.5,1,1),"cm"))

Load in data for just fish taxa and reformat

HeatMethods <- read.csv("HTSvsHP_HeatChart_FishPrey.csv", header = T)
HeatMethods$Group <- as.character(HeatMethods$Group)
HeatMethods$Group <- factor(HeatMethods$Group, levels=unique(HeatMethods$Group))
HeatMethods$Group <- factor(HeatMethods$Group, levels = rev(levels(HeatMethods$Group)))
summary(HeatMethods)
meltedHeatMethods <- melt(HeatMethods)

Plot heat chart for just fish taxa

ggplot(meltedHeatMethods, aes(variable, Group)) +
 geom_tile(stat = "identity", aes(fill = value), colour = "white") +
 scale_fill_gradientn(na.value = "white", colours=viridis(4, direction = -1),
 values=rescale(c(1,2,3,4)),
 breaks = c(1,2,3,4),
 labels = c("Only molecular","Only morphological",
 "Molecular and morphological at same taxonomic level",
 "Molecular and morphological at different taxonomic
 level"), guide = "legend") +
 theme(legend.position="none",
 axis.title.x = element_blank(),
 axis.title.y = element_blank(),
 axis.text.x = element_blank(),
 axis.text.y = element_text(size=12, colour = "black"),
 plot.margin=unit(c(0.5,0.5,1,1),"cm"))

Load in data for non-fish taxa and reformat

HeatMethods2 <- read.csv("HTSvsHP_HeatChart_AltPrey.csv", header = T)
HeatMethods2$Group <- as.character(HeatMethods2$Group)
HeatMethods2$Group <- factor(HeatMethods2$Group,

levels=unique(HeatMethods2$Group))
HeatMethods2$Group <- factor(HeatMethods2$Group,

levels = rev(levels(HeatMethods2$Group)))
summary(HeatMethods2)
meltedHHeatMethods2<- melt(HeatMethods2)

Plot heat chart for just fish taxa

ggplot(meltedHHeatMethods2, aes(variable, Group)) +
 geom_tile(stat = "identity", aes(fill = value), colour = "white") +
 scale_fill_gradientn(na.value = "white", colours=viridis(4, direction = -1),
 values=rescale(c(1,2,3,4)),
 breaks = c(1,2,3,4),
 labels = c("Only molecular","Only morphological",
 "Molecular and morphological at same taxonomic level",
 "Molecular and morphological at different taxonomic
 level"), guide = "legend") +
 theme(legend.position="none",
 axis.title.x = element_blank(),
 axis.title.y = element_blank(),
 axis.text.x = element_blank(),
 axis.text.y = element_text(size=12, colour = "black"),
 plot.margin=unit(c(0.5,0.5,1,1),"cm"))

NMDS spider plot of different methods

method <- read.csv(**“**Lorna Otter HP-HTS NMDS_onlydoubles.csv")

method$Sample <- as.factor(method$Otter)

method$Method <- as.factor(method$Method)

methodnmds <- method[,3:(ncol(method)-1)]

method.mds <- metaMDS(comm = methodnmds, distance = "jaccard**"**, trymax=999, k=2, trace = FALSE, autotransform = FALSE)

plot(method.mds$points); text(method.mds, row.names(method.mds))

method.mds$stress

scrs <- scores(method.mds, display = 'sites')

scrs <- cbind(as.data.frame(scrs), Sample = method$Sample, Method = method$Method)

cent <- aggregate(cbind(NMDS1, NMDS2) ~ Sample, data = scrs, FUN = mean)

segs <- merge(scrs, setNames(cent, c('Sample', 'oNMDS1', **'**oNMDS2')), by = 'Sample', sort = FALSE)

methodpal <- brewer.pal(8, "Set1")

methodpal

methodpiplot <- ggplot(scrs, aes(x = NMDS1, y = NMDS2, fill = Method)) +

scale_fill_manual(values=methodpal) +

geom_point(alpha=0.5, size = 3, pch = 21) +

geom_segment(data = segs, mapping = aes(xend = oNMDS1, yend = oNMDS2), alpha=0.4) +

theme_bw()

methodpiplot

**S2.4** **Model based analysis of dietary data**

Load in data and check format
HTSHP2 <- read.csv("HTS+HPThreePlusNoInsectORMollusc.csv", header = T)
summary(HTSHP2)
colnames(HTSHP2)
rownames(HTSHP2) <- HTSHP2[,1]
rownames(HTSHP2)
str(HTSHP2)

Create object of only species consumed

dietHTSHP2 <- mvabund(HTSHP2[,21:47])
Create model and check aasumptions
MVdietHTSHP2 <- manyglm(dietHTSHP2 ~ Sex + Size + Size:Sex + Scored.SMI +

Year2 + Season + Sex:Season + Size:Season + long + lat + lat:long +

KmRiverDist + WaterClass + Urban + Sex:WaterClass + Size:WaterClass +

KmRiverDist:Sex + KmRiverDist:Size , family = binomial(link="cloglog"),

data = HTSHP2)
plot(MVdietHTSHP2)

Conduct model simplification by stepwise deletion by AIC and extract signifcance of variables on the general prey composition and specific prey groups

step(MVdietHTSHP2, test = "Chisq")
MVdietHTSHP2.2 <- manyglm(dietHTSHP2 ~ Season + long + KmRiverDist
 , family = binomial(link="cloglog"), data = HTSHP2)
plot(MVdietHTSHP2.2)
anovaMVdietHTSHP2.2 <- anova(MVdietHTSHP2.2, resamp = "montecarlo",

test = "LR", p.uni="adjusted")
anovaMVdietHTSHP2.2

**S2.5** **Bayesian Ordination And Regression Analysis (BORAL)**

Create an object with only significant variables from the mvabund analysis and then run BORAL with and without this object

X <- as.data.frame(HTSHP2[,c(9,16,20)])
dietHTSHP2Boral <- boral(dietHTSHP2, family = "binomial", lv.control=list(num.lv=2))
dietHTSHP2Boral2 <- boral(dietHTSHP2, X=X,family = "binomial",

lv.control=list(num.lv=2))

Check BORAL objects and plot ordinations (first need to run the lvsplot2 function code; O’Hara *et al.* 2016)

summary(dietHTSHP2Boral)
plot(dietHTSHP2Boral)
lvsplot(dietHTSHP2Boral, return.vals = T)
lvsplot2(dietHTSHP2Boral, alpha=0.5, main="", cols.lvs = "grey78", cols.coefs =

"springgreen4", a=1.3, jitter = TRUE)
summary(dietHTSHP2Boral2)
plot(dietHTSHP2Boral2)
lvsplot(dietHTSHP2Boral2, return.vals = T)
lvsplot2(dietHTSHP2Boral2, alpha=0.5, main="", cols.lvs = "grey78", cols.coefs =

"springgreen4", a=1.3, jitter = TRUE)

**S2.6** **Bipartite plots**

Load in data and check format

BipartiteHTSHP <- read.csv("Combined_HTS_HP_Bipartite.csv", header = T)
summary(BipartiteHTSHP)
rownames(BipartiteHTSHP) <- BipartiteHTSHP[,1]
rownames(BipartiteHTSHP)
colnames(BipartiteHTSHP)

*Plot data for season*

plotweb(BipartiteHTSHP[,3:6], text.rot=90,
 col.high = c("darkolivegreen3", "gold2", "indianred2", "steelblue3"),
 bor.col.high = c("darkolivegreen3", "gold2", "indianred2", "steelblue3"),
 col.interaction = c("darkolivegreen3", "gold2", "indianred2", "steelblue3"),
 bor.col.interaction = c("darkolivegreen3", "gold2", "indianred2", "steelblue3"),
 bor.col.low = "ivory4", col.low = "ivory4", y.width.low = 0.1, high.xoff = F,
 low.y = 0.7, high.y = 1.7, labsize = 2.3, low.spacing = 0.0175,
 high.spacing = 0.15, method = "normal", text.low.col = "black")

*Plot data for longitude*

plotweb(BipartiteHTSHP[,7:272], text.rot = 90, col.high = viridis(263),
 bor.col.high = viridis(263), method = "normal", col.interaction = viridis(263),
 bor.col.interaction = viridis(263), high.lablength = 0, high.spacing = 0.00145,
 low.spacing = 0.015, low.y = 0.7, high.y = 1.7, text.low.col = "black",
 labsize = 2.3)

*Plot data for distance from the coast*

plotweb(BipartiteHTSHP[,273:531], text.rot = 90, col.high = viridis(256),
 bor.col.high = viridis(256), method = "normal", col.interaction = viridis(256),
 bor.col.interaction = viridis(256), high.spacing = 0.00145, low.spacing = 0.015,
 low.y = 0.7, high.y = 1, text.low.col = "black", labsize = 2.3)

**Appendix S3: Comparison of model outputs for both dietary analysis methods**

To demonstrate the consequences for ecological interpretation by using morphological and molecular methods together, compared to using each in isolation, the primary multivariate model within the manuscript was repeated as described in the main text, but with molecular and morphological data in isolation. The morphological data was run in two separate models: one in which data were aggregated as for the main model with the exclusion of poorly resolved cyprinid and salmonid identifications (hereafter ‘morphological model 1’), and again with these detections represented as simply ‘salmonid’ and ‘cyprinid’ (hereafter ‘morphological model 2’).

Using molecular data alone, dietary composition significantly related to longitude (MGLM: LRT Deviance = 62.4, p = 0.002) and river distance (MGLM: LRT Deviance = 79.1, p = 0.001), with bullhead (MGLM: LRT Deviance = 12.263, p = 0.019) and brown trout (MGLM: LRT Deviance = 18.372, p = 0.002) occurrences specifically related to river distance. In morphological model 1, otter dietary composition significantly related to season (MGLM: LRT Deviance = 45.50, p = 0.008), water class (MGLM: LRT Deviance = 108.19, p = 0.008), longitude (MGLM: LRT Deviance = 68.59, p = 0.002), river distance (MGLM: LRT Deviance = 44.29, p = 0.049) and interactions between otter sex and otter size (MGLM: LRT Deviance = 69.16, p = 0.025), water class and otter sex (MGLM: LRT Deviance = 83.95, p = 0.003), and otter size and river distance (MGLM: LRT Deviance = 87.98, p = 0.004). Specifically, bullhead occurrences related to water class (MGLM: LRT Deviance = 17.291, p = 0.026), eel occurrences to the interaction between otter sex and otter size (MGLM: LRT Deviance = 18.548, p = 0.031), and goby occurrences to season (MGLM: LRT Deviance = 11.707, p = 0.017). In morphological model 2, otter dietary composition significantly related to season (MGLM: LRT Deviance = 72.09, p = 0.013), water class (MGLM: LRT Deviance = 92.65, p = 0.002), longitude (MGLM: LRT Deviance = 55.87, p = 0.001), and interactions between otter sex and otter size (MGLM: LRT Deviance = 59.74, p = 0.018) and otter sex and water class (MGLM: LRT Deviance = 85.55, p = 0.001). Specifically, pike occurrences significantly related to longitude (MGLM: LRT Deviance = 19.845, p = 0.005), and eel occurrences to interactions between both otter sex and otter size (MGLM: LRT Deviance = 18.846, p = 0.025), and otter sex and water class (MGLM: LRT Deviance = 29.799, p = 0.003).

The results of the model based on the molecular data more closely resembled those of the combined analysis from the main text. There were, however, some commonalities with the results of the morphological analysis too, demonstrating that this combined approach represents the variation present in both datasets. The disparate ecological conclusions reached by using each dataset alone highlights the risk in basing ecological analyses and management decisions on single-method studies, but also the strength in combining approaches.
